# Multi-product biorefineries from lignocelluloses: a pathway to revitalisation of the sugar industry?

**DOI:** 10.1186/s13068-017-0761-9

**Published:** 2017-04-11

**Authors:** Somayeh Farzad, Mohsen Ali Mandegari, Miao Guo, Kathleen F. Haigh, Nilay Shah, Johann F. Görgens

**Affiliations:** 1 Department of Process Engineering, Stellenbosch University, Stellenbosch, 7600 South Africa; 2 Department of Chemical Engineering, Imperial College London, London, SW7 2AZ UK

**Keywords:** Biorefinery, Biofuel, Biochemical, Techno-economic evaluation, Life cycle assessment (LCA), Sugarcane residues, Multi-products

## Abstract

**Background:**

Driven by a range of sustainability challenges, e.g. climate change, resource depletion and expanding populations, a circular bioeconomy is emerging and expected to evolve progressively in the coming decades. South Africa along with other BRICS countries (Brazil, Russia, India and China) represents the emerging bioeconomy and contributes significantly to global sugar market. In our research, South Africa is used as a case study to demonstrate the sustainable design for the future biorefineries annexed to existing sugar industry. Detailed techno-economic evaluation and Life Cycle Assessment (LCA) were applied to model alternative routes for converting sugarcane residues (bagasse and trash) to selected biofuel and/or biochemicals (ethanol, ethanol and lactic acid, ethanol and furfural, butanol, methanol and Fischer–Tropsch synthesis, with co-production of surplus electricity) in an energy self-sufficient biorefinery system.

**Results:**

Economic assessment indicated that methanol synthesis with an internal rate of return (IRR) of 16.7% and ethanol–lactic acid co-production (20.5%) met the minimum investment criteria of 15%, while the latter had the lowest sensitivity to market price amongst all the scenarios. LCA results demonstrated that sugarcane cultivation was the most significant contributor to environmental impacts in all of the scenarios, other than the furfural production scenario in which a key step, a biphasic process with tetrahydrofuran solvent, had the most significant contribution.

**Conclusion:**

Overall, the thermochemical routes presented environmental advantages over biochemical pathways on most of the impact categories, except for acidification and eutrophication. Of the investigated scenarios, furfural production delivered the inferior environmental performance, while methanol production performed best due to its low reagent consumption. The combined techno-economic and environmental assessments identified the performance-limiting steps in the 2G biorefinery design for sugarcane industry and highlighted the technology development opportunities under circular bioeconomy context.

**Electronic supplementary material:**

The online version of this article (doi:10.1186/s13068-017-0761-9) contains supplementary material, which is available to authorised users.

## Background

A range of environmental concerns, e.g. fossil fuel depletion and climate change, have triggered development of the bio-based economy, where biorefineries and bio-products are key features [1]. Various government initiatives have been launched at international, national and regional levels to support the biofuel, bioenergy and other biochemical production, such as mandated biofuel blending targets in the United States (USA), Brazil, Canada and several EU member states [2–6]. The biofuels strategy in South Africa (SA) targets at a 2% penetration of liquid biofuels in the national fuel market, aiming to boost the rural economy and create “green” jobs [7]. To date, investments in biofuels in South Africa have been very modest. However, conversion of 70% of the total estimated biomass availability would be sufficient to meet 24% of the SA liquid fuel needs as bioethanol equivalent [7]. Besides, bio-based chemicals including non-food starch, cellulose fibres and cellulose derivatives, tall oils, fatty acids and fermentation products have been also regarded as important components of the bio-based economy [8–10].

Amongst a range of potential bio-based resources, lignocellulosic biomasses (the so-called second-generation or 2G biomass) especially waste 2G feedstocks including agroprocessing residues (e.g. sugarcane bagasse) are promising resources for production of bio-based fuels and chemicals, due to their relative abundance, potential lower supply costs and avoidance of land use competition with food crops [11, 12]. Considerable potential 2G waste biomass is generated annually in SA (26 million tonnes per annum including agricultural and forestry residues and invasive plants), and provides a significant opportunity for the SA biofuels/biochemical industry as well as potential for rural economy development [13]. Such 2G waste resources and related economic sectors can play a significant role in a potential circular bioeconomy, contributing to a resource-efficient sustainable SA future.

South Africa along with other BRICS countries (Brazil, Russia, India and China) represents the emerging market and growing world economy in the coming decades. BRICS countries dominate the global sugar market [14]. SA has the largest sugar industry in African continent, making substantial contribution to the development of national economy [15]. However, the global sugar industry generally is facing a range of issues such as the fluctuating and low global sugar prices, increasing competition with low-cost global sugar producers, increasing energy and agricultural input costs and ageing facilities [16]. More efficient use of 2G sugarcane residues to broaden the product range of sugar mills has been proposed as a potential solution [13]. Citing SA as an example, about 270–280 kg bagasse are generated per tonne of harvested cane, which is primarily used inefficiently as boiler fuel in current sugar mills [17]. Approximately 7.6 million tonnes of bagasse is generated in South Africa annually [18], which potentially could lead to a $1.5 billion bioethanol market [19].

Biorefineries are regarded as the cornerstone of a bioeconomy, where a range of thermochemical and biochemical routes including non-catalytic or catalytic technologies can be deployed and integrated to transform biomass organic molecules to a plethora of bio-products [20]. In the context of sugar industry, a biorefinery could be annexed to a sugar mill to increase diversification and support regional and rural development. A number of economic assessments have been published on biofuel production from 2G waste biomass, considering biochemical [21, 22] or thermochemical technologies [23–26]. Production of value-added chemicals and polymers from lignocellulosic waste biomass has been also explored in the past few years [10, 27–29]. Research and development continue to be necessary on economic and environmental aspects of co-producing biochemicals along with biofuel.

Apart from economic viability, environmental impact is another important factor underpinning sustainable development of biorefinery systems. A widely recognised environmental impact assessment tool is life cycle assessment (LCA) [30], which is a cradle-to-grave evaluation approach formalised by the International Organization for Standardization [31]. LCA has been widely applied in biorefinery technologies [32–34], but the majority of the studies focused on GHG emissions and energy balances with less attention paid to the wider range of environmental impact categories [35]. Apart from environmental and economic aspects, biorefinery can contribute to poverty reduction in rural areas through job creation and increased income for small farmers [36].

A review of literature suggests that very limited research has been conducted on the combined LCA and economic assessments, to compare alternative technological approaches to biofuel and biochemical production from lignocellulosic waste biomass. The overall objective of this work is to assess the holistic impacts of diversification in the sugarcane industry through the development of lignocellulosic biorefineries, in terms of economic viability, environmental and social benefits of using 2G waste resource as feedstock. In this regard, six potential biochemical/thermochemical pathways—bioethanol, bioethanol and lactic acid, bioethanol and furfural, butanol, methanol and Fischer–Tropsch synthesis—have been investigated (see Table 2 for the justification of these choices) to explore sustainable biorefinery design. South Africa sugarcane system is used as a representative case study in our modelling research to identify the performance-limiting steps in the biorefinery design and highlight the future technology development opportunities. Overall, this study aims to provide scientific insights into the biorefinery ‘plug-in’ (annexed) solutions to existing sugar industry that could contribute best to the sustainability and circular bioeconomy development under the BRICS emerging economy context.

## Methods

### Sugarcane 2G biomass availability

The 2G sugarcane residues, which could be utilised as biorefinery biomass, include bagasse (currently inefficiently burned at sugar mills) and brown leaves (currently burned in the fields) [18, 37]. As demonstrated in Table 1, the biomass availability [65 tonnes dry mass per hour (tDM/h)] has been calculated based on the average data in South Africa. It is assumed that only the brown leaf component of sugarcane harvesting residues will be made available by a green-cane-harvesting approach, while the tops (green leaves) will be left in the field to maintain soil fertility [18, 38]. Such harvesting approach represents South Africa cane trash recovery system, which has been discussed in details in previous studies [18].

**Table 1:** Detail of available feedstock for a typical South African sugar mill

Material	Percentage	t/h	Reference
Sugarcane	–	300	[18, 39, 40]
Wet bagasse	30% of sugarcane	90	[18, 40]
Dry bagasse^a^	50% of wet bagasse	45	[18, 39, 41]
Total tops and trash	15% of Sugarcane	45	[18]
Trash available for biorefinery	50% of total residue	22.5	[18, 41]
Dry trash^a^	85% of wet residue	~20	[18, 39, 41]
Total feedstock^a^ DM/wet		65/113	

^a^Extracts are included in dry matter (DM)

In this study, the average chemical composition of bagasse and brown leaves of SA sugar mills has been used, i.e. 40.7% cellulose, 27.1% hemicellulose, 21.9% lignin, 3.5% ash and 6.7% extractive and total water content of mixture 42% (dry base) [42].

### Scenarios description

Via a thorough review of potential biochemical and thermochemical pathways, six fuels/chemicals were selected based on potential application, technology maturity and market demand (Table 2). Methanol and Fischer–Tropsch (FT) liquids were investigated through the thermochemical conversion of lignocellulose to biofuels, as an alternative to biological conversion [43]. Six scenarios were included in this study, i.e. 2G bioethanol: baseline technology (scenario 1), co-production of lactic acid with ethanol (scenario 2), furfural and ethanol (scenario 3), butanol (scenario 4), methanol (scenario 5) and Fischer–Tropsch syncrude (scenario 6). In all scenarios, surplus electricity was produced after satisfying the operational demands of the combined sugar mill and annexed biorefinery, and was assumed to be a co-product exported to grid.

**Table 2:** Selected bio-products in the bio-based market. Adopted from [52]

	Potential application	Volume	Sales	% of total market	Reference
		(1000 t/year)	(M$/year)		
Ethanol	Dominant biofuel, globally	71,310	58,141	93	[44–46]
*n*-Butanol	Replacement of petroleum-derived butanol	590	1115	20	[47]
Furfural	Platform chemical, conversion to petro-chemicals	300–700	300–1015	100	[48]
Lactic acid	Multiple commodity, i.e. acrylic acid, 1,2-propanediol, pyruvic acid	472	684	100	[49–51]

The selected biorefinery scenarios were developed under the assumption that they would be annexed to an existing South African sugar mill, where representative conditions of a typical SA sugar mills was considered. The whole system was assumed to be self-sufficient in energy, i.e. the operational energy requirement for both the sugar mill and adjacent biorefinery was met exclusively by the available lignocellulosic biomass or components thereof, without any supplementary fossil energy input. Sugarcane bagasse from the mill combined with brown leaves was simulated as the feedstock for the biorefinery, for both the Combined Heat and Power (CHP) plant and the biofuel/biochemical conversion processes. The CHP plant was assumed to replace the inefficient boiler in the existing sugar mill, providing steam and electricity to both the sugar mill and annexed biorefinery.

All of the scenarios were used to develop detailed process models covering all the key unit operations and simulated in detail using Aspen Plus^®^ (Aspen Technology Inc., USA), which allows rigorous definition of processes, equipment and utility requirements [53]. The assumptions used for simulation of biorefinery scenarios are presented in Tables S1 and S2 of Additional file 1.

#### Scenario 1: production of ethanol and electricity

**Fig. 1:**
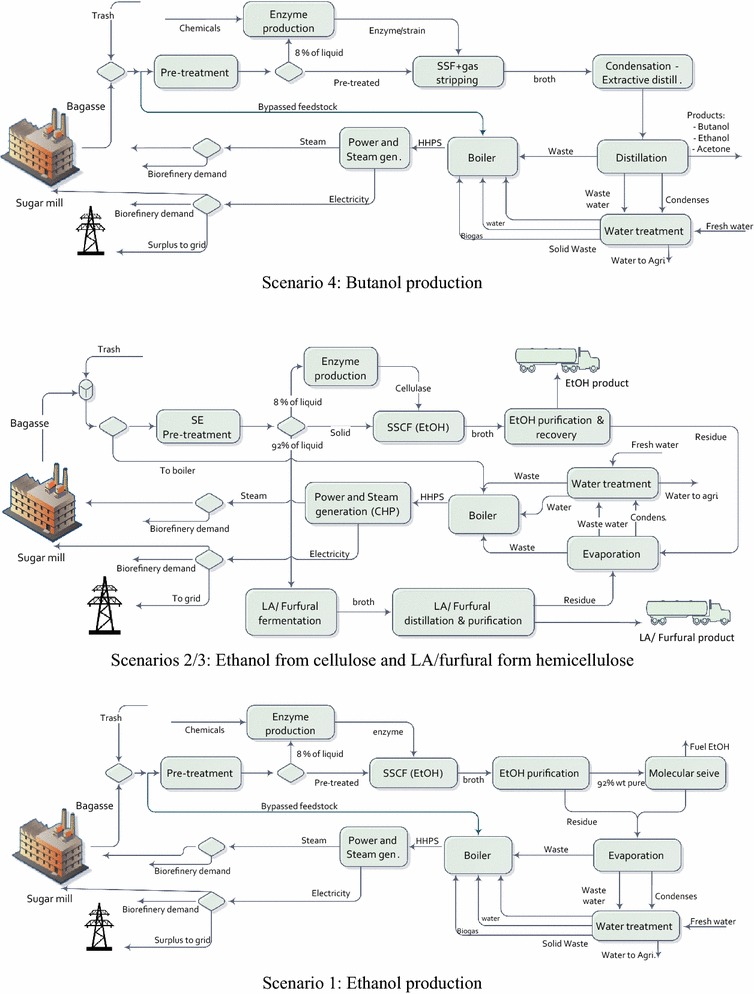
Block flow diagram of the biochemical scenarios

Steam explosion pretreatment, catalysed with sulphur dioxide (SO_2_), along with simultaneous saccharification and co-fermentation (SScF) was adopted in the process model for ethanol production, based on the promising conversion performance demonstrated in previous studies [54–57]. To minimise enzyme supply costs [58], an on-site cellulase enzyme production unit was modelled in the biorefinery simulation, based on the literature data sourced from the National Renewable Energy Laboratory [59]. The purification and recovery section was modelled to separate water, anhydrous ethanol and combustible solids from the fermentation broth. The produced ethanol is purified up to 92.55% wt using two distillation columns followed by a molecular sieve dehydration column to meet fuel grade ethanol (99.5% wt purity) [60–63]. The evaporation unit was simulated not only to purify stillage water for internal recycling, but also to produce a syrup of solubles suitable for co-feeding to the boilers, along with solid residues [64–66]. Water treatment unit (Fig. 1) includes waste water treatment (WWT) and water cycle for CHP unit. In WWT, during anaerobic digestion biogas is produced and sent to CHP unit, while the treated water is recycled internally [57].

#### Scenario 2: production of ethanol, lactic acid and electricity

The scenario for producing lactic acid from lignocellulosic biomass included four main steps [67], i.e. pretreatment, enzymatic hydrolysis, fermentation and separation–purification. Along with the ethanol derived from the cellulose portion, lactic acid is simulated as a product from the hemicellulose fractions as illustrated in Fig. 1. These processes share a single pretreatment unit, after which the sugar streams are divided according to aforementioned bioprocesses. During the fermentation process, the produced lactic acid is continuously neutralised using Mg(OH)_2_, to minimise its inhibitory effect to the fermenting strains, forming Mg-lactate. The latter was assumed to react with a water-miscible organic amine, trimethylamine (R_3_N), forming Mg(OH)_2_ crystals and a R_3_N-lactate complex, in an exchange reactor. The Mg(OH)_2_ crystals were filtered and recycled back to fermentation; the R_3_N-lactate complex was thermally decomposed to release lactic acid and the R_3_N was recycled back to the exchange reactor [68, 69]. Purification of crude lactic acid product from fermentation broth [70], in a two-step reaction process, involved the esterification of lactic acid and hydrolysis of the ester, giving the highest purity levels (99.5% wt) [71, 72].

#### Scenario 3: furfural, ethanol and electricity production

In scenario 3, processes for co-generation of furfural, bioethanol and electricity were simulated, where bioethanol and furfural are derived from the cellulose and hemicellulose components, respectively [73, 74] using similar pretreatment and ethanol production processes as scenario 2 (lactic acid production; Fig. 1). In the presence of hydrochloric acid catalyst, the hemicellulose fraction obtained from pretreatment is converted to furfural, which is followed by the addition of tetrahydrofuran (THF) for furfural extraction and side reaction prevention in a two-phase reaction–separation system [48, 74, 75]. The organic solid fractions were separated from the aqueous phase by a downstream decanter, and sent to the distillation stage for product recovery. THF is separated and recycled back after the first and second furfural distillation columns, and the mixture of formic acid and acetic acid are extracted from the rest, through the third distillation column. The fourth column produces furfural with 99.8% wt purity, while the fifth column is applied to separate formic acid and acetic acid as minor side products [73].

#### Scenario 4: co-production of butanol and electricity

The acetone–butanol–ethanol (ABE) fermentation [76, 77] using strains of the bacterium, *Clostridia*, under anaerobic conditions, was modelled for butanol production from both hexose and pentose sugars [78, 79]. Dilute acid pretreatment was selected for scenario 4, based on available experimental data in the literature, rather than steam explosion pretreatment applied for ethanol production in scenarios 1, 2 and 3 [80–82]. To avoid feed and product inhibition, simultaneous saccharification and fermentation (SSF) combined with gas stripping was selected for hydrolysis–fermentation of pretreated lignocellulose, where the medium for gas stripping and mixing in the fermenters was modelled as fermentation gases (CO_2_ and H_2_) [82]. The ABE products are recovered from the gas by condensation at 0 °C via a cryogenic cycle, followed by further purification [82, 83]. Liquid–liquid extraction was simulated to separate butanol, ethanol and acetone from the condensate, followed by conventional distillation to purify butanol to 99.5% wt (main product), along with ethanol and acetone (side products) [84].

#### Scenario 5: production of methanol and electricity

**Fig. 2:**
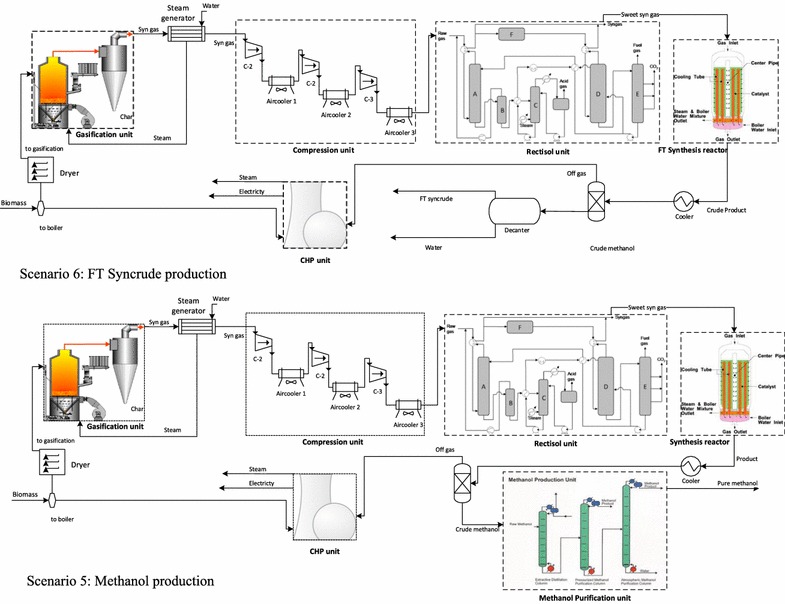
Block flow diagram of the thermochemical scenarios

Allothermal gasification (ALO-G) was modelled for syngas production from sugarcane lignocelluloses [85], in which the heat for the endothermic gasification reactions was assumed to be supplied by a heat transfer medium, such as a bed material circulated between combustion and gasification chambers in a dual fluidised bed gasifier [86–88]. The gasification conditions for very low-tar syngas production were simulated [85]. Separated from the bed material after gasification process [89], the syngas undergoes cooling (Fig. 2), and conditioning, where CO_2_ and H_2_S are removed from the syngas in the Rectisol unit [90, 91]. Once cleaned and conditioned, the syngas is compressed to 98 bar (liquid phase) and converted to methanol in a fixed bed synthesis reactor [92]. The reactor feed is pre-heated to the reaction temperature of 250 °C, which is isothermally maintained by steam generation, while cooling of the reactor effluent is achieved by heating up the reactor feed directly through a double fluid heat exchanger. The vapour phase product from the single-pass synthesis reactor is cooled down to recover the methanol and separate unconverted syngas and inert gaseous species (CO_2_, CH_4_) [93]. The combined gas streams are heated before expansion through a turbo expander generator to recover some of the compression energy of the gas by generating electricity [85].

#### Scenario 6: production of syncrude and electricity via Fischer–Tropsch synthesis (FTS)

Under scenario 6, the conditioned syngas derived from scenario 5 was assumed to be converted to Fischer–Tropsch (FT) syncrude in a low-temperature reactor [94]. Under an advanced FT synthesis process, high-pressure reactor conditions allow the wax products existing in liquid phase (with catalyst suspended) to be removed from the bottom of the reactor. Since the reaction heat is rapidly dispensed through the liquid medium to a cooling medium, a syngas conversion of up to 90% per pass is possible [95]. An equal distribution between the four primary hydrocarbons product types (i.e. gases, naphtha, diesel and waxes) was assumed [96], with an occurrence probability of 90%, following the trends of Anderson–Schulz–Flory [94].

### Economic assessments

An economic assessment was implemented using Aspen Plus^®^ Economic Evaluator and literature data to estimate the purchased and installed cost of equipment and variable and fixed operating costs, based on technical information obtained from process simulations [97]. Internal rate of return (IRR) was adopted as the indicator of investment feasibility. The operational costs were categorised as variable operating costs (the cost of feedstock, chemicals and disposal waste) and fixed operating costs (fixed cost of the plant independent of production capacity, including labour costs, maintenance, and property insurance and tax) [97]. The economic parameters specific to South Africa were selected for the economic evaluation in this study and are presented in Table 3.

**Table 3:** Parameters of the economic analysis, based on South Africa

Parameter	Value	Reference
Working capital (% of FCI)	5%	[97, 99]
Depreciation period (years)	25	[97]
Depreciation method	Straight line	
Salvage value	0	
% Spent in year 0	100%	
Income tax rate	28.0%	(http://www.sars.gov.za)
Cost year for analysis	2015	
Inflation rate	5.7%	(http://www.resbank.co.za)
Operating hours (h/year)	6480^a^	[37]
Currency convertor USD $ 1 = ZAR	14.0	(http://www.xe.ir)
Min. acceptable IRR (nominal)	15%	(http://www.resbank.co.za)
Chemical engineering plant cost index (CEPCI)	490.6	Extrapolation of data [97]
Base prices (year 2015)		
Ethanol price ($/L)	0.596	(http://www.energy.gov.za)
Lactic acid ($/t)	2000	(http://www.nnfcc.co.uk)
Furfural ($/t)	1200	[52]
Butanol ($/t)	1000	(http://www.energy.gov.za)
Methanol ($/L)	0.43	(http://www.methanex.com)
Syn crude ($/US gallon)	1.3^b^	(http://www.oil-price.net)
Trash price ($/t)	53.2^c^	(http://www.indexmundi.com)
Bagasse ($/t)	0^d^	
Electricity price ($/kWh)	0.08^e^	(http://www.ipprenewables.co.za)

^a^It is function of sugar mill operating hours (9 months)

^b^Since 60% of syncrude is used to produce gasoline (Brent price −5 $/bbl) and the rest is used for diesel production (Brent price +6 $/bbl), syncrude price was assumed the same as crude oil price

^c^The price of brown leaves (trash) was estimated based on the unit price of coal and heating value of trash in proportion to coal plus collection cost to the framers [18, 100]

^d^The price of bagasse is assumed as zero and in return the thermal and electrical power required for sugar mill operation is supplied by CHP unit of biorefinery

^e^Electricity price is assumed to be slightly higher than current industrial electricity price “green electricity” which represents some government financial support of biorefineries. This is realistic for the SA scenario, where there is a greater commitment from government to support green electricity rather than biofuel

The cost of the bagasse from the sugar mill was assumed to be of equivalent economic value as the steam and electricity provided back to the sugar mill by the CHP plant. It worth noting that subsidies and direct financial support from government were excluded from the analyses under the current study as a stringent approach to identify opportunities that are economically viable for expanding the bio-sector under the developing country context, where less governmental financial support would be available compared with the well-subsidised biosectors in the developed world [7, 98].

### LCA methodologies

#### Model systems and functional unit

**Fig. 3:**
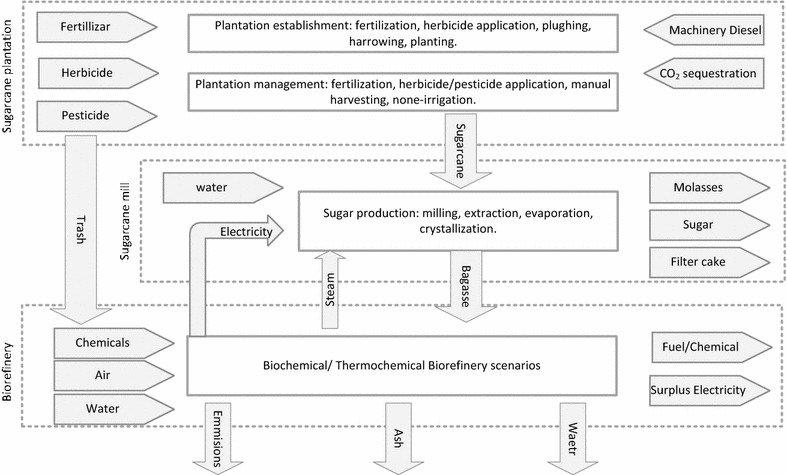
System boundary for investigated biorefinery scenarios

In this study, an attributional life cycle assessment (aLCA) approach was applied to evaluate the environmental viability of 2G biorefinery designs for the conversion of sugarcane residues to a range of fuels and biochemicals via thermochemical and/or biochemical routes. As illustrated in Fig. 3, production pathways for bioethanol, biobutanol, lactic acid, furfural, biomethanol, syncrude derived from sugarcane bagasse and trash are included in the LCA system boundary. The LCA system boundary (Fig. 3) includes production of investigated products, i.e. bioethanol, methanol, lactic acid, etc., from sugarcane bagasse and trash, as well as sugarcane cultivation, harvesting, transportation and bagasse production in the sugar mill (the residue of sugar production process).

The LCA study aimed to provide insights into the environmentally sustainable pathways for sugarcane residue utilisation and ‘hot-spot’ identification (i.e. main environmental contributors) for each cradle-to-biorefinery-gate system. The functional unit was defined as “a biorefinery with the capacity of processing 65 (tDM/h) tonnes bagasse and trash per hour”.

#### Allocation approach

Based on ISO guideline [101], different allocation approaches have been considered. Under the current study, allocation procedure is not avoidable for unit-processes generating multiple co-products, e.g. sugar mill producing sugar, molasses, bagasse and filter cake. Moreover, allocation by physical relationships (either mass or energy content) cannot reflect the underlying relationships between the co-products under an economic value-driven multi-product system. Thus for multi-output systems, an economic allocation approach was used as a default method to partition the input–output flows and environmental burdens according to the respective value and quantity of co-products. An alternative allocation approach recommended by EU Renewable Energy Directive [102]—energy allocation, where the environmental burdens were allocated amongst the co-products (e.g. bioethanol and lactic acid) based on their energy contents—was applied in the sensitivity analysis.

A stoichiometric carbon-counting approach was used to ‘track’ the carbon flows from sugarcane residue into bio-products over the cradle-to-biorefinery-gate life cycle. Other carbon flows (e.g. fermentation emissions, etc.) were assumed to be as CO_2_ and were therefore treated as carbon-neutral. This C-counting approach with regard to the bio-products was applied to determine (1) carbon ‘sequestration’ into the bio-products (from the sugarcane cultivation phase of the life cycle) and (2) downstream release of this carbon during the subsequent processing stages of the bio-product life cycle. The sequestration of carbon into biomass during the cane growth phase thus represents a ‘negative’ GHG emission at this life cycle stage but this carbon could then be returned to the environment in various ways depending upon the subsequent fate of the products. In current study, we assumed soil carbon as steady state, i.e. no soil carbon accumulation or change in mid-term due to the carbon-neutral balance between below-ground biomass degradation and CO_2_ emissions from soil organic matter turnover. However, the below-ground soil carbon turnover and the carbon cycling in cane–soil ecosystems can be simulated using biogeochemical modelling approach, which is driven by climate, soil properties, planation management and anthropogenic activity [103, 104]. Such detailed simulation can be included in future studies. A schematic diagram of carbon flows through a representative biorefinery is depicted in Figure S1 of Additional file 1.

#### Life cycle inventory, impact assessments and data quality analysis

Complete inventories for the life cycle of sugarcane residue-derived bio-products were developed by combining simulation results of advanced 2G biorefineries (in particular, detailed material and energy balances) and literature data representing sugarcane cultivation in South Africa and sugar mill processing technology [105, 106]. The inventory of sugarcane cultivation and sugar milling is presented in Tables S5 and S6 of Additional file 1, respectively.

Life cycle impact assessment (LCIA) methodologies can be categorised as midpoint- and endpoint-oriented approaches which are also termed as ‘problem-oriented’ and ‘damage approach’, respectively. The former is chosen along with environmental mechanisms between the life cycle inventory (LCI) results and endpoints [107] and the latter is defined at the level of protection area [108]. Two midpoint approaches developed by the Centre of Environmental Science (CML) of Leiden University and Technische Universitat Berlin, respectively—CML-IA baseline 3.02 and water scarcity [109]—were applied as default characterisation methods in this study at the LCIA stage where the evaluation focused on 12 impact categories, viz. abiotic depletion, global warming potential (GWP100), acidification, eutrophication, ozone depletion (ODP) and photochemical oxidation (POCP), terrestrial, aquatic eco-toxicity and human toxicity, water consumption and water scarcity. Damage-oriented approaches were applied to sensitivity analyses, i.e. Eco-Indicator 99 hierarchist version (EI 99H/A) and pfitzer Eco-indicator 99 (Water footprint) defining impact categories at the endpoint level. A scenario sensitivity analysis method was used to examine the sensitivity of the LCA findings to the LCIA methodological choice [110]. A 10% change in the characterised LCIA profiles for a single product system or a reversal of the rank order of the LCA comparisons was chosen as sensitivity thresholds above which the influence of LCIA method was considered to be significant. The LCA model was implemented in Simapro 8.0.4.30 (PRe Consultants 2014).

## Results and discussion

### Overall mass and energy balance

The overall mass and energy balances (heating, cooling and power demand) of the simulated biorefinery scenarios are presented in Table 4, where the bypass of the feedstock required for the CHP unit varied with the energy balances of individual scenarios. These were in agreement with previous reports, showing the need to combust a portion of the unprocessed lignocellulose feedstock to meet the energy demands of the integrated sugar mill-biorefinery processes [111]. The highest bypass requirement (58 wt%) was for the co-production of furfural and ethanol, which can be attributed to the high steam demand for separating THF from the reaction mixture and the significant amount of THF inputs as feed in the two-phase production system.

**Table 4:** Overall mass and energy balance of the studied biorefinery scenarios

	Unit	Scenario 1	Scenario 2	Scenario 3	Scenario 4	Scenario 5	Scenario 6
		EtOH	EtOH-LA	EtOH-Fur.	Butanol	Methanol	FT syncrude
Feedstock							
Bypass to boiler	t/h	22.75	26.00	32.50	26.00	22.75	19.50
	%	35.00	40.00	50.00	40.00	35.00	30.00
To biorefinery	t/h	42.25	39.00	32.25	39.00	42.25	45.50
Products							
Ethanol	t/h	11.00	7.48	5.66	0.35	–	–
Lactic acid	t/h	–	4.65	–	–	–	–
Furfural	t/h	–	–	2.07	–	–	–
Butanol	t/h	–	–	–	4.61	–	–
Methanol	t/h	–	–	–	–	12.76	–
Syncrude	t/h	–	–	–	–	–	5.80^e^
Acetone	t/h	–	–	–	1.50	–	–
Acetic acid	t/h	–	–	1.25	–	–	–
Surplus elc.	MW	7.10	5.60	7.50	4.30	0.50	1.80
Total production^a^	t/h	11.00	12.13	9.22^c^	6.46	12.76	5.80
	t/t^d^	0.26	0.31	0.29	0.16	0.30	0.13
Energy demand^b^							
Cooling	MW	50.70	45.50	52.00	73.10	26.20	33.90
Heating	MW	69.10	66.60	66.30	61.70	2.60	2.40
Power	MW	2.00	2.00	2.30	13.70	14.20	13.70

^a^Total production of chemicals

^b^Heat and power demand of sugar mill is excluded

^c^Produced formic acid is included

^d^Production yield: tonne of product(s) per tonne of biomass fed to biorefinery “exclusive of feedstock bypassed to CHP”

^e^Density of syncrude = 634.8 kg/m^3^

**Fig. 4:**
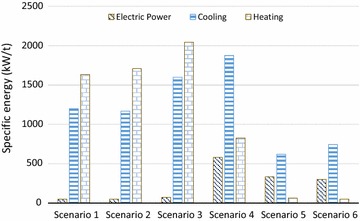
The specific energy consumption (kW per tonne feedstock to biorefinery) of the studied biorefinery scenarios—sugar mill demand is excluded—(scenario 1 ethanol; scenario 2 ethanol, LA; scenario 3 ethanol, furfural; scenario 4 butanol; scenario 5 methanol; scenario 6 FTS; Detail data are presented in Table S3 of Additional file 1)

FT synthesis demonstrated the highest lignocellulosic biomass availability for process conversion (70% of the total feed; 30% bypass), primarily benefiting from the exclusion of the energy costs of syncrude purification in scenario 6. The energy requirement per unit of biomass is presented in Fig. 4 for all the scenarios. Butanol production showed the highest electrical power and cooling demands due to the cryogenic requirements of the gas-stripping process, while furfural was the most energy-intensive process particularly with heating demand, because of the steam requirement for furfural recovery and purification.

As represented in Table 4, scenario 2 demonstrated a superior total production rate to scenario 1 but lower electricity generation. It can be ascribed to the fact that the reaction of sugars to ethanol is a hetero-fermentative process with CO_2_ as by-product, while LA production from sugars is homo-fermentative process without carbon-containing by-products [112]. As a result, during fermentation of the liquid fraction after pretreatment, xylose was converted to LA with a higher conversion (91% of the theoretical maximum of 1 g/g) [49], than xylose to ethanol (76.6% of the theoretical maximum of 0.51 g/g). Scenario 3 showed better biomass conversion efficiency than scenario 1 [0.29 and 0.26 (t/t), respectively], due to a more favourable xylose conversion pathway to furfural (85%) [73] than to ethanol (76.6%) [113]. However, a lower total biochemical production rate (92.2 t/h) was observed in scenario 3 in comparison with scenarios 1 and 2, because of its low proportion of biomass available for biorefinery processes after meeting CHP needs (high lignocellulose bypass to the boiler).

Due to the less efficient conversion from glucose and xylose to butanol (56 and 20%, respectively [114]) compared to ethanol, furfural and lactic acid production, scenario 4 turned out to be an inferior system with the lowest biochemical production rate amongst all simulated routes.

Although the production yield of methanol (scenario 5) and ethanol-LA (scenario 2) was broadly similar (0.302 and 0.311 t/t, respectively), scenario 5 showed a higher biochemical production (12.76 vs. 11 t/h), but significantly lower electricity outputs (0.5 vs. 7 MW) than scenario 1. This could be explained by (1) the large amount of unconverted hydrocarbon residues (lignin and unconverted cellulose and hemicellulose) and the resulting high power export potential for biochemical processes [39], compared to the thermochemical routes, where both the lignin and carbohydrate components of lignocelluloses are converted to fuels/chemicals products, and (2) high electrical power demands for operating compressors and air-cooler fans (syngas purification) in gasification scenarios [99].

Methanol production represented the superior thermochemical route with better technical performance than FT synthesis, which was mainly driven by their different overall biomass conversion rates. Although the same processes were modelled for syngas production and conditioning in scenarios 5 and 6, the energy content of methanol fuel from syngas was 37.1% higher than for the FT synthesis [85]. This could be explained by the fundamental reactions for methanol (CO + 2H_2_ → CH_3_OH) and FT liquid production (CO + 2H_2_ → –CH2– + H_2_O), which demonstrate that the water formation in FT synthesis results in a loss of chemical energy [115]. In addition, biomethanol and FT syncrude differ in their fuel quality and applicability as drop-in biofuels. Mixtures of methanol and gasoline have been already launched in the market. In China, national fuel blending standards of M85 (85% methanol, 15% gasoline) and M100 (100% methanol) has been developed since 2009, and M15 standard is in progress [116, 117]. Therefore, produced methanol can be used as drop-in fuel. While the produced syncrude needs more deoxygenation and upgrading processes to derive different fuels, i.e. diesel, gasoline, jet fuel [85, 118], it cannot be used as fuel in the current format. Such biofuel quality aspect is out of current study scope but can be further discussed in future research.

### A comparison of the economic viability

**Fig. 5:**
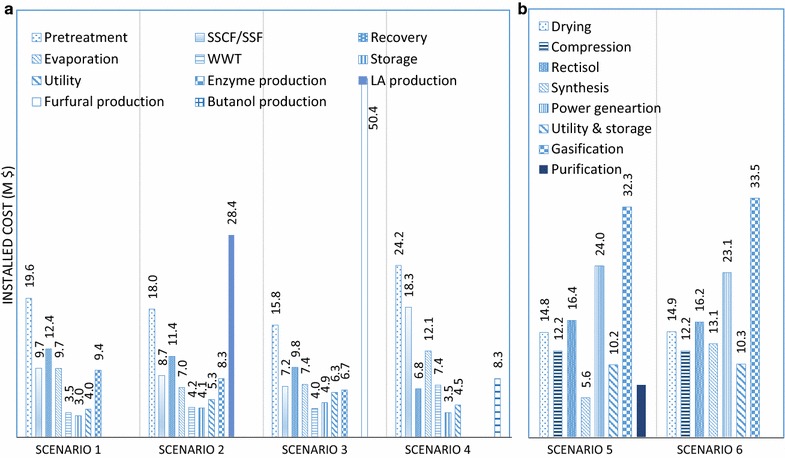
Detailed installed cost of the biochemical biorefinery scenarios (CHP is excluded which costs $61–63.1 million) (**a** biochemical scenarios, **b** thermochemical scenarios)

The calculated total capital investment (TCI), operating costs (OPEX), total sales and IRR for all the scenarios are presented in Table 5. The installed costs of the biochemical biorefinery scenarios excluding the CHP plant are given in Fig. 5.

**Table 5:** Concise results of economic evaluation for studied scenarios

	TCI (million $)^a^	Fixed operating cost (million $/year)	Variable operating cost (million $/year)^b^	Total sales (million $/year)^b^	IRR %
Scenario 1	240	8.1	10.2	58.6	14.7
Scenario 2	288	9.5	24.4	99.5	20.5
Scenario 3	321	10.8	16.0	54.1	7.5
Scenario 4	269	5.8	17.2	40.2	4.8
Scenario 5	233	8.5	7.9	56.5	16.7
Scenario 6	234	8.1	8.2	12.6	11.5

^a^Total Capital Investment (TCI). The boiler and power generation sections contributing approximately 20–25% of the TCI ($60–64 million)

^b^First year of economic analysis (2015)

The simulations presented in the current study were regarded as representative of industrial scale production. The bioethanol production capacity of scenario 1 [90.4 million litre per year (ML/year)] compared well with commercial lignocellulosic bioethanol plants (e.g. Abengoa plant with capacity of 90 ML/year [119]), whereas the estimated TCI of $2.66/L also agreed with the reported industrial data $2.64/L (Abengoa Hugoton Project, USA; http://www.abengoa.com ), $2.65/L Beta Renewables (Italy; http://www.betarenewables.com), $2.52/L Iogen Costa Pinto Project (Brazil; http://www.iogen.ca). The simulation of the biomethanol scenario was comparable with the ongoing commercial scale plants such as Varmlands Methanol in Sweden (production capacity: 92,000 t/year, TCI: $290 million) and Woodsprit Projects in Netherlands (production capacity: 413,000 t/year, TCI: $550 million) [120].

Comparison of scenarios 1, 2 and 3 indicated that the required TCI may increase with product diversification. The TCIs of the ethanol production sections in scenarios 2 and 3 were lower than scenario 1 (ethanol only), due to diversion of the hemicellulose component to lactic acid or furfural production. However, additional capital expenses were incurred for the production of lactic acid and furfural (scenarios 2 and 3), increasing the total capital cost by $48 million (20.0% of TCI) and $61 million (25.4% of TCI), respectively. The furfural reactor is particularly expensive, due to the high pressure (55 bar) and acid corrosion accounted for in the design of the two-phase system. We note that integration of first- and second-generation (1G2G) fermentation-based technology for bioethanol production has been demonstrated to be cheaper than a standalone 2G biorefinery [121]. However, evaluation of the 1G2G scenarios for ethanol and lactic acid production was not in the scope of this study (in all cases the sugar products are not converted further) and will be presented in future work.

Our analyses demonstrated research findings which are consistent with previous studies, i.e. butanol production by fermentation is economically uncompetitive and technically challenging compared to bioethanol production [3, 122]. Furthermore, dilute acid pretreatment is more energy intensive than steam explosion [123], thus requiring a larger energy supply, which resulted in a higher TCI for butanol production than ethanol production.

The TCI of the gasification scenarios, measured by installed costs [approximately $123 million, including the CHP plant (Fig. 5b)], was lower than the biochemical processes [installed cost ranges from $132 to 174 million; Fig. 5a). Previous reports have also indicated that some thermochemical pathways may be economically more favourable than the biochemical routes [39, 124], with the advantage of ability to accommodate more a diverse range of biomass and the avoidance of large water flows in many process steps [125]. However, a comparison study of bioethanol production through biochemical and thermochemical pathways revealed that the biochemical route is presently the leading process strategy in the US and EU [119], whereas the overall economics of the two processes are broadly similar [121, 126, 127]. Determination of the most desirable pathway between thermochemical and biochemical conversion routes has been quite controversial. In general, bioethanol production through biochemical pathway seems the more preferred route in US, while the EU projects are almost equally distributed between biochemical and thermochemical conversion platforms [128]. Thermochemical conversion processes have been ordered according to increasing lowest capital cost, i.e. hydrogen, methanol, lignocellulosic ethanol and FT diesel [129]. However, in this study FT synthesis was simulated for syncrude production rather than diesel, gasoline or jet fuel, thus leading to similar TCIs for the gasification scenarios. In this context, the better performance of methanol production over the FT synthesis was partly due to better market price of methanol over syncrude, with the price of the latter closely linked to crude oil.

**Fig. 6:**
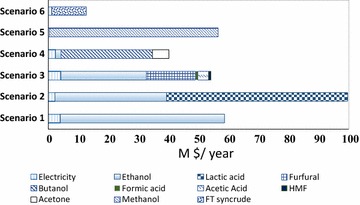
Detailed sales of the studied scenarios based on the products (relevant data are given in Table S4 of Additional file 1)

Scenarios 2 and 5 were found to be the most economically profitable scenarios, considering the revenue (Fig. 6). As a result of a higher methanol production rate compared to ethanol, scenario 5 had a higher revenue than scenario 1, but lower than scenario 2 (61% of its revenue derived from LA). Of all the products considered, electricity delivered the lowest contribution to the revenue (less than 5%). Furfural co-production with ethanol did not improve the economic viability of the baseline biorefinery as it has lower total sales compared to scenario 1, with 30% of revenue derived from furfural and 53% from the ethanol sales. As stated in other studies, modern furfural commercial production is inefficient (25–35 tonne steam consumption per tonne of produced furfural) and suffers from low yields (<50 mol% of theoretical) [48]. This was avoided to some extent in our simulations and analyses by selection of an indirect, two-phase system for co-production of furfural and ethanol from lignocelluloses. Further research efforts should be placed to improve furfural yields, including the removal of furfural into the vapour phase, the extraction of furfural from the catalytically active aqueous phase using biphasic solvent systems and the application of reusable or recoverable solid catalysts [48]. Alternatively, scenarios for direct furfural production from lignocellulose, with co-production of ethanol from solid residues, should be simulated in the future, pending availability of experimental data to describe the performance of these processes.

The ethanol-LA (scenario 2; IRR 20.5%) and methanol synthesis (scenario 5; IRR 16.7%) were the only scenarios with an IRR greater than the minimum expected return on investment of 15% in South Africa. Amongst all scenarios, the synthesis of methanol represented a promising system with reasonable investment potential (high IRR combined with low TCI). Scenario 2 demonstrated that an increase in the investment potential for cellulosic ethanol production could be achieved by co-producing lactic acid, whereas scenario 3 showed reversed trends with decreased investment potential by incorporating furfural co-production into bioethanol biorefineries.

### Sensitivity analysis for economic viability

**Fig. 7:**
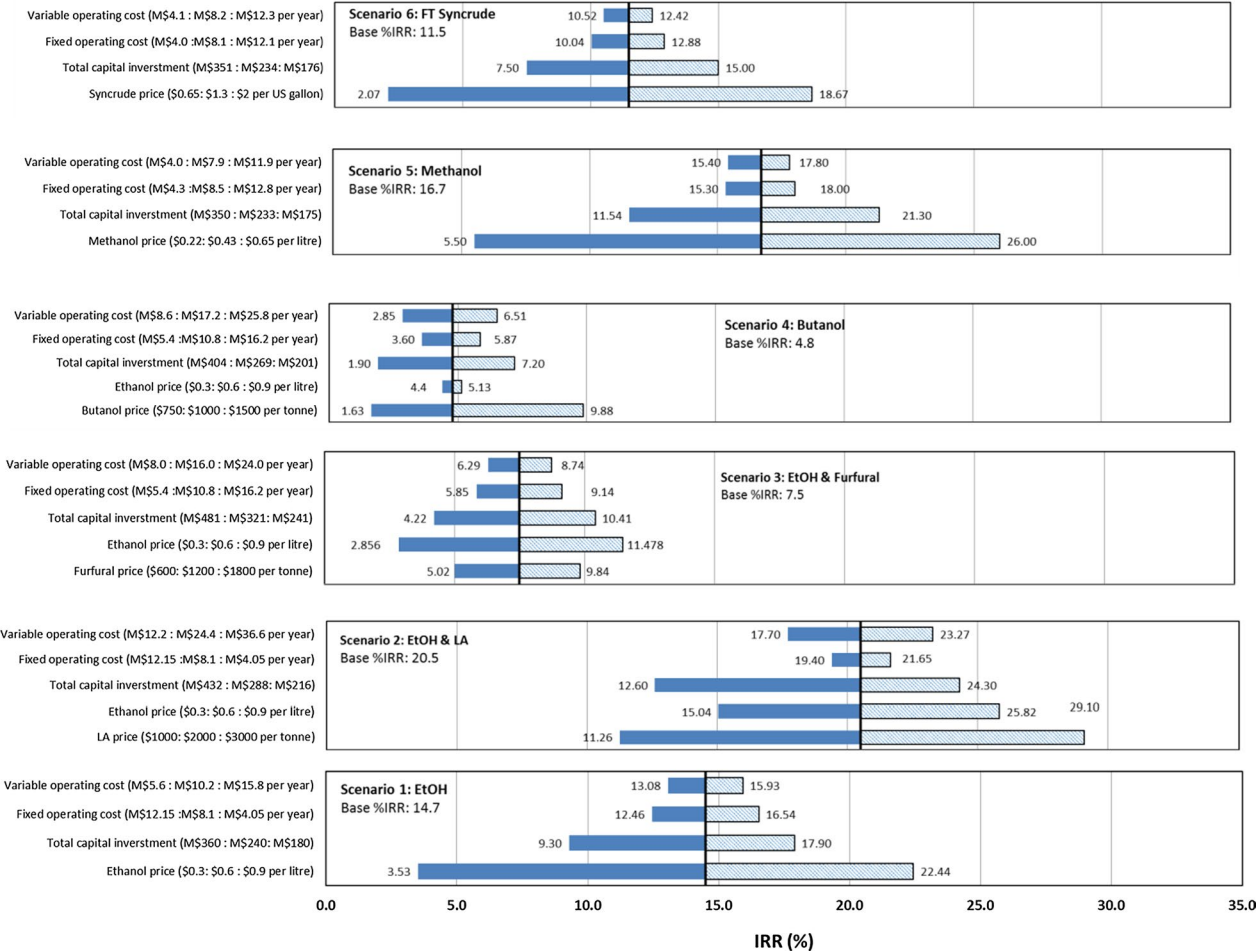
Results of economic sensitivity analysis of the studied biorefineries

A sensitivity analysis was conducted to test how sensitive the economic viability is to variability in input data. The electricity price was eliminated from the sensitivity analysis due to its insignificant contribution to revenue, whereas the price of brown leaf as a harvesting residue was accounted for in the analyses as an embedded factor in the variable operating cost. As given in Fig. 7, the product prices, together with the fixed operating costs and TCI, produced the most significant impacts on the economic evaluation.

The results of the sensitivity analysis (Fig. 7) indicated that product price had the most significant effect on IRR values, with scenarios 5, 6 and 1 showing the highest sensitivity to product price, respectively. The profitability of scenario 6 was sensitive to FT syncrude price (estimated based on crude oil data) as a 25% increase in syncrude price leads to a profitable case. However, such a finding represents a conservative estimation due to the current low oil price.

As demonstrated in Fig. 7, another key parameter for IRR was TCI; scenarios 2 and 5 can meet the minimum profitability criteria (IRR of 15%) with 25% increase in the TCI. In general, variable and fixed OPEX were not key factors, although the feedstock cost have an exceptional impact on the economic viability of biorefineries [130–132]. In the annexed biorefinery case studies, the bagasse is represented as an internal material flow without counting its traded price (other than value of steam and electricity supplied back to the sugar mill at no internal cost), while only the trash price was accounted for in the OPEX. Overall, scenarios 2 and 5 presented robust cases, with a 25% decrease in the lactic acid price or a 50% drop in ethanol price; scenario 2 was still profitable; scenario 5 retained profitability potential with a 20% reduction in the methanol price. Generally, polygeneration leads to a more flexible scenario with regard to changes of market demand [133].

The revenue of the furfural production scenario is largely driven by the ethanol price (53% revenue from bioethanol sales), leading to a case with low investment potential; it can be economically viable only if the ethanol price increases by approximately 75%, or a significantly lower-cost production method is selected. Conversely, the investment potential of scenario 2 is not influenced by the ethanol revenue, but rather by lactic acid, which is caused by the dominance of lactic acid income (61%) in the total revenue.

### Environmental evaluation

**Fig. 8:**
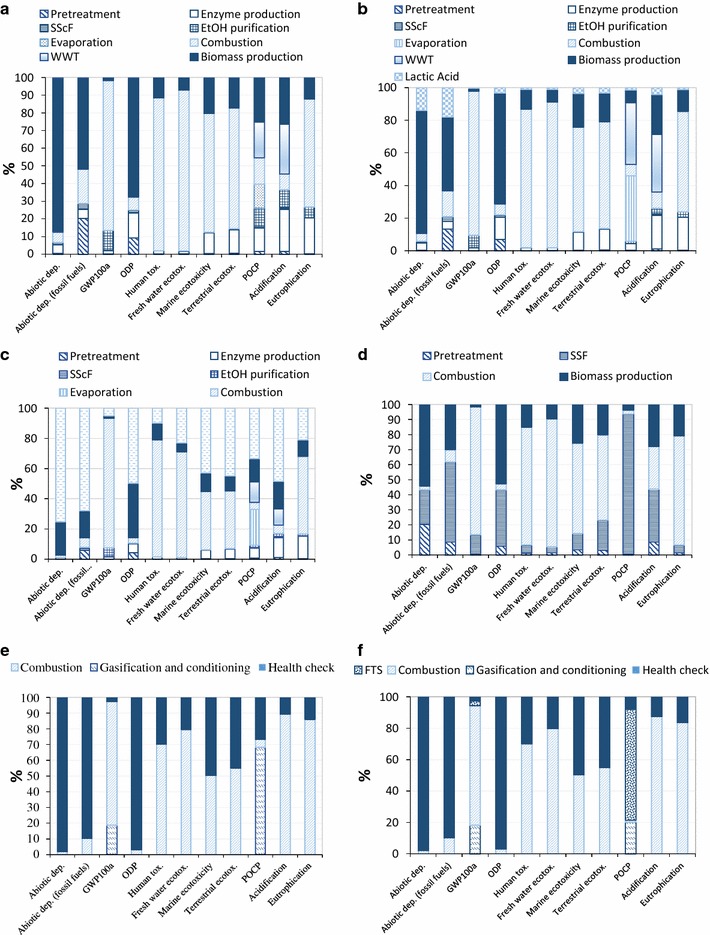
Characterised LCIA profiles of biorefineries; (unit: 1 tonne of product as defined in the caption; Method CML-IA 2 baseline). **a** Scenario 1 ethanol; **b** scenario 2 lactic acid; **c** scenario 3 furfural; **d** scenario 4 butanol; **e** scenario 5 methanol; **f** scenario 6 FT syncrude

**Fig. 9:**
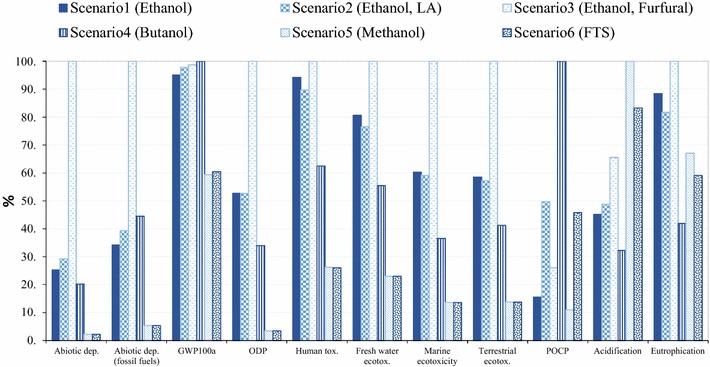
Characterised LCIA profiles for comparison of biorefinery scenarios (including biogenic C, method: CML-IA baseline 2)

**Fig. 10:**
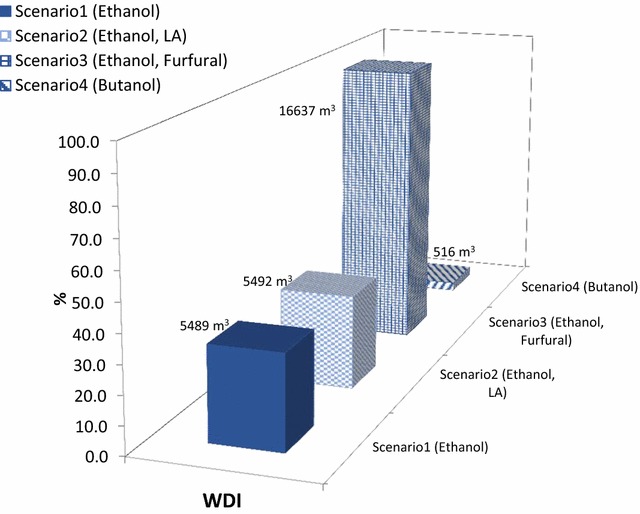
Water consumption of investigated scenarios per hour

**Fig. 11:**
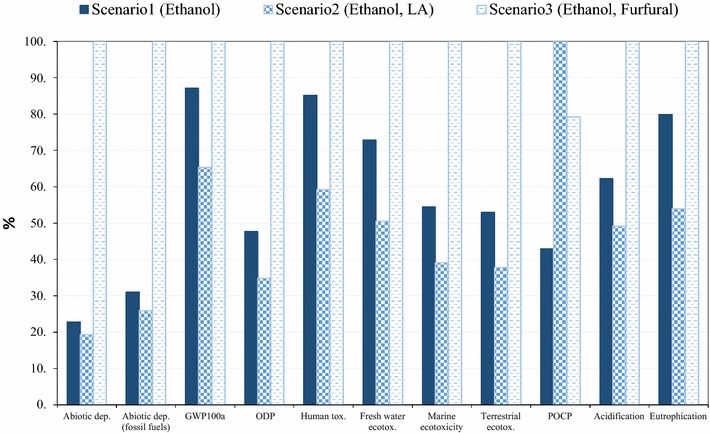
Comparison of different scenarios for production of 1 tonne bioethanol (method: CML-IA baseline)

**Fig. 12:**
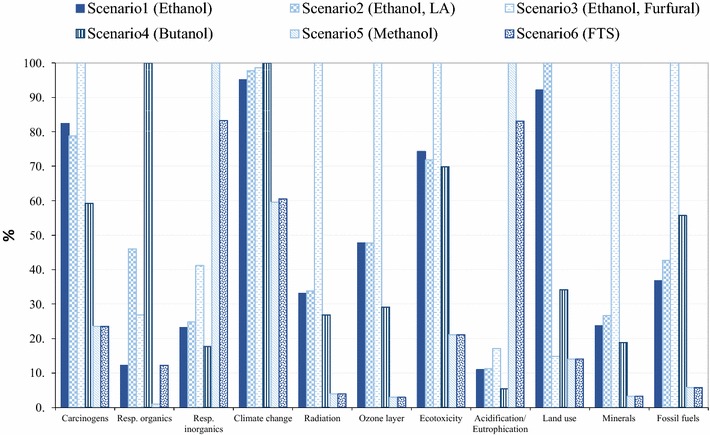
Characterised LCIA profiles of investigated biorefineries (method: EI 99H)

The results for all LCA impact categories and normalised comparisons (%) are presented in Figs. 8, 9, 10, 11, 12. The LCIA scores for each individual impact category and scenarios are given in Additional file 1 (Tables S8–S16).

#### Cradle-to-biorefinery-gate LCA contribution analysis

The environmental profiles of biofuel and biochemical production via different scenarios are given in Fig. 8. The results implied that the environmental benefits of bio-based products varied significantly with scenarios. In general, the main contributors to environmental impacts were agrochemical production (herbicides, N/P fertilisers, diesel consumption) and combustion stages (emissions and ash released) in all of the scenarios, except for scenario 3, where furfural production stage (THF, NaCl and HCl consumption) dominated the environmental impacts. The chemical production processes, which varied with scenarios, were important drivers of the differences between the environmental profiles of biorefinery scenarios. However, since the unit of bagasse processed in biorefinery has been defined as the functional unit, agricultural inputs were broadly similar within scenarios. Nitrogen fertilisers played an important role in the LCIA impacts of biomass cultivation, which resulted in NO_*x*_ and NH_3_ emissions during fertiliser production and consequently contributed to acidification and eutrophication environmental categories [134].

As demonstrated in Fig. 8a for the bioethanol production (scenario 1), the biomass (sugarcane cultivation and bagasse production in the sugar mill) contributed about 65–85% to abiotic depletion, fossil fuel depletion and ODP impacts, while it delivered 3–35% of the environmental impacts across all other impact categories due to agrochemical inputs in plantation and diesel consumption as well as emissions released from agricultural lands (i.e. N leaching). The abiotic depletion is dominated by the fossil fuel oil consumption during the harvesting process and transportation of sugarcane. The combustion unit in scenario 1 not only contributed 83–88% of the environmental burdens in GWP_100_, and eco-toxicities impact categories, but also led to 61% eutrophication impacts because of flue gas emissions (CO_2_, CH_4_, No_*x*_) produced by the biomass combustion. Landfilling of ash from combustion caused 70% of impacts on human toxicity due to heavy metals such as Antimony released to waterborne bodies [135, 136]. Enzyme production and combustion units were the main contributors to eutrophication impacts (20 and 61%, respectively) due to waterborne emissions such as phosphate caused by consumption of phosphate fertilisers and diammonium phosphate (for enzyme production). The WWT unit contributed 35% in acidification and POCP, due to airborne emissions such as SO_2_. About 10–12% of the impacts on GWP_100_ and POCP burdens were attributed to flue gas emitted to the atmosphere during bioethanol production and purification, e.g. N_2_, CO_2_ and SO_2_ released as well as ethanol loss.

Similar to scenario 1, sugarcane cultivation accounting for 20–75% of the environmental burdens on acidification, ODP, eutrophication and abiotic depletion, was the main cause of environmental issues in scenario 2 (Fig. 8b). The biomass combustion stage contributed to about 35% of acidification and POCP impacts due to airborne emissions such as SO_2_ caused by urea consumption. However, lactic acid production was also one of the main contributors to environmental burdens as a consequence of chemical consumption [K_2_HPO_4_, MgSO_4_, trimethylamine (TEA)]. The LA production process accounted for 5–26% of abiotic depletion, acidification, human toxicity as well as eutrophication, due to phosphate fertiliser and magnesium sulphate consumption. It contributed to 40% of POCP, mainly because of the ethanol released to the atmosphere in LA production process.

Considering scenario 3 (Fig. 8c), the furfural production process was the main environmental contributor, accounted for 33–75% of burdens in POCP, ODP, acidification, abiotic depletion (fossil fuel) as well as abiotic depletion due to emissions to air (e.g. CH_4_, C_2_H_6_ and SO_2_) and heavy metal released to water (e.g. chromium), as a consequence of HCl, NaCl and THF consumption during the process. In eutrophication, furfural production caused 21% of impacts due to THF and HCl consumption, whereas the main contributor was biomass combustion (51%) due to phosphate released to water as a consequence of phosphate fertiliser inputs at the biomass cultivation stage.

As demonstrated in Fig. 8d, the environmental burdens were mainly attributable to sugarcane cultivation, the combustion stage and the SSF unit in scenario 4. The SSF unit contributed to 30–50% of total impacts on acidification, ODP and abiotic depletion due to emissions to atmosphere, i.e. nitrogen oxides and methane (as a consequence of urea consumption and transport). This unit showed a significant contribution to POCP burdens (93%), due to ethanol, butanol and acetone emissions to the atmosphere.

In gasification scenarios (Fig. 8e, f), biomass gasification contributed around 20% of GWP_100_ and POCP environmental burdens as a consequence of considerable CO_2_ and CO emission during the syngas conditioning process. The combustion stage represented a dominating contributor (range of 50–90%) to the impacts on GWP_100_ (because of CO_2_ emissions), human toxicity (as a result of heavy metals released to water bodies such as Antimony), acidification and eutrophication (due to the emission of nitrogen oxide to air). The FTS contributed to less than 5% of GWP_100_ burdens and over 70% POCP impacts as a result of CH_4_, CO and butane release to the atmosphere.

Previous studies indicated that a 10% increase in the biomass output and its availability (per unit land area) for biorefinery processing incurred more CO_2_ savings than a 10% increase in the yield of the bio-conversion methods [30]. This is consistent with our research finding from the contribution analyses above, which suggested that improvements in agrochemical utilisation efficiency in agricultural practice (e.g. lower fertiliser inputs per unit biomass harvested) would lead to overall environmental savings particularly on abiotic depletion and ODP. In addition, further research efforts should be placed on the chemical reduction in biochemical routes and the effective emission abatement technology and ash disposal at combustion stage.

#### LCA comparison

In order to identify the sustainable scenarios for utilisation of biomass, the LCIA of the investigated biorefinery pathways based on the conversion of a functional unit of feedstock (65 tDM/h) has been compared as presented in Fig. 9.

The results for LCIA comparisons varied with impact categories. Overall, the gasification scenarios (thermochemical pathway) benefitted from their lower chemical inputs and energy demands; therefore, they presented environmental advantages over biochemical pathways on most of the categories, except for acidification and eutrophication. Indirect emissions associated with process chemical consumption have been reported as the hot spot of the biochemical pathway, while the thermochemical route has been determined to have lower GHG emissions in previous studies [121, 137, 138]. However, our gasification scenarios incurred some higher burdens, for example, due to acidification due to the NO_*x*_ released from the combustion of biomass.

The gasification scenarios showed broadly similar environmental performances in most of the impact categories, because syngas production and conditioning processes did not vary with scenarios. However, scenario 5 represented slightly higher acidification and eutrophication impacts than scenario 6, as a consequence of the higher bypass (35% in scenario 5 vs. 30% in scenario 6) of the feedstock to the CHP. On the other hand, scenario 6 had a three-time higher POCP environmental burden than scenario 5, due to emissions from the FTS process, i.e. methane and butane emissions which do not occur in methanol production process.

Comparing the biochemical pathways, butanol delivered a better performance than other routes in most of the categories because of lower chemical inputs, while it suffers from higher POCP impacts compared with other scenarios, due to the ethanol and acetone emissions released from the distillate stage. The co-production of ethanol and furfural (scenario 3) presented the worst biochemical scenario on most of the environmental impact categories. This can be explained by the highest feedstock bypass in scenario 3, which resulted in significantly higher GHGs and eutrophication emissions, as well as high consumption of chemicals (such as THF, HCl and NaCl) leading to higher environmental burdens in the ODP, abiotic depletion and acidification impact categories. Scenarios 1 and 2 represented similar environmental outcomes in abiotic depletion, acidification, GWP_100_ and ODP, while scenario 2 incurred higher POCP and acidification burdens than scenario 1. That can be ascribed to the slightly lower bypass in scenario 1 (35 via 40%) and higher chemical inputs in LA process (K_2_HPO_4_ and TEA).

Water use is categorised into required water for the processes, while sugarcane cultivation in South Africa is mostly non-irrigated. As reflected in Fig. 10, furfural production (scenario 3) has the highest water consumption profiles, which was mainly driven by the waste water treatment section.

The environmental burdens of the three scenarios producing ethanol (scenario 1–3) were compared to identify the most sustainable pathway to produce bioethanol (Fig. 11). The results implied that scenario 2 delivered the best environmental performance in almost all the impact categories, except for POCP, where additional ethanol emissions during the LA production process (consuming ethanol) in scenario 2 resulted in a higher POCP burden than the other two scenarios. Scenario 1 produced a greater amount of unconverted residue than scenario 2, which is sent for combustion in CHP unit, and thus resulted in a less favourable option for bioethanol production than scenario 2, whereas scenario 3 is presented as the least sustainable bioethanol production system due to the consumption of chemicals such as THF and HCl in furfural production.

#### Sensitivity analysis on characterisation model and allocation approach

As an alternative to the midpoint method CML-IA baseline, the damage-oriented method Eco-Indicator 99 H (hierarchist, version 2.10) was applied to the LCA model. Although the impact categories evaluated in the two methods are not identical, most of them overlapped. The CML-IA baseline represents eco-toxicity in three sub-categories, while Eco-Indicator 99H shows only an aggregated eco-toxic indicator result. As an equivalent to photochemical oxidation in CML-IA baseline (summer smog), Eco-indicator 99 includes a respiratory organics impact category where respiratory effects resulting from exposure to organic compounds in summer smog are evaluated [139, 140]. Eco-indictor 99 also accounts for winter smog (respiratory inorganic), damages induced by radioactive radiation and conversion and occupation of land [139] all of which are not in the scope of CML baseline method. Unlike the CML method, EI 99 aggregates acidification and eutrophication potential of all substances into a single indicator result. Abiotic depletion in CML accounts for the aggregated mineral resources, which is equivalent to the mineral impact category under EI99 method, whereas both CML fossil abiotic depletion and EI99 fossil fuel impact categories focus on fossil fuel resources only.

As demonstrated in Fig. 12, the gasification scenarios appear to deliver the highest impact in the aggregated acidification/eutrophication EI 99 category, while according to the CML findings, the gasification scenarios incurred higher acidification, but lower eutrophication scores compared to biochemical scenarios. The results of EI 99 broadly agreed with CML method in abiotic depletion, fossil fuel depletion, GWP_100_, eco-toxicity, POCP and ODP impact categories.

### Socio-economic impacts and overall sustainability assessment

Regardless of the emphasis placed on environmental aspects of biofuel, many authors have agreed on the key role of socio-economic motivations in the rise of biofuel markets. Biorefineries can provide significant job creation, mostly in the supply of feedstock to the sugar mill, i.e. the harvesting residues. The so-called “green cane harvesting” as an alternative to burning cane before harvesting would essentially double the number of jobs in harvesting, while also creating additional jobs in collection and transport of residues to the sugar mills. Based on the available data on South African and Brazilian agriculture [18, 140], the created job for manual trash collection from the field, baling the material and transportation was estimated as 0.205 t/man-h. All the scenarios were assessed processing the same amount of feedstock (145,800 t/year); therefore, the number of created jobs for green harvesting, collection and transformation was estimated as 89,000 man-day/year for all the scenarios [141, 142]. The number of jobs created at the process stage was estimated based on the complexity of the processes as 48, 60, 60, 40, 43 and 39, respectively, for the evaluated scenarios (1–6). Due to high levels of automation applied in processing plants, the number of skilled jobs created, including plant manager, engineers, supervisors, operators and administrative jobs, was limited [143].

**Fig. 13:**
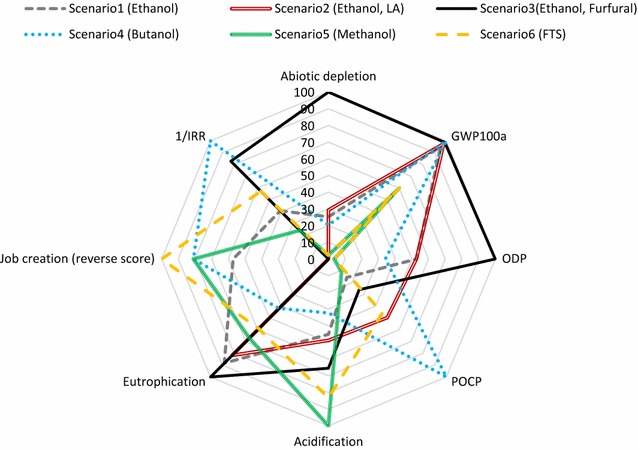
Sustainability analyses of the investigated scenarios

A normalised spider chart has been introduced in this study to illustrate the different pathways with eight indicators, i.e. IRR, job creation, eutrophication, GWP_100_, ODP, POCP, acidification and abiotic depletion. The route with the largest occupied area represents the inferior system. The results of the sustainability assessment (Fig. 13) imply that scenario 5 outperformed the other case studies, benefitting from its environmentally superior profile. The scenario 2 (lactic acid production) delivered the highest IRR, better socio-economic sustainability and a lower GWP_100_ score than other biochemical routes, based on its IRR and job creation potential. Furfural production (scenario 3) appeared as the least economically competitive and environmentally friendly option in comparison with other routes, although it exhibits good job creation potential. To achieve better sustainability scores in scenario 3, research and development attention should be placed on minimising the solvent consumption (or increasing solvent internal recycling) and improving the furfural productivity. Our research also suggested a range of bottlenecks for further improvement of the overall performance in the scenarios with biphasic production systems particularly achieving the trade-offs between environmental sustainability and economic viability, i.e. solvent recycling which has potential for reduction in environmental damages and solvent costs but may incur additional operational costs.

## Conclusions

Detailed modelling, simulations and analyses of six scenarios have been developed to gain a better understanding of the potential for product diversification in the sugarcane industry by means of biorefineries. All the biorefinery scenarios were designed on the basis of being self-sufficient in bioenergy, thus rerouting a portion of the total lignocellulose feedstock directly to the boiler (CHP plant), rather than supplementing bioenergy sources with fossil fuels such as coal or natural gas. Economic evaluation of the scenarios implied that scenarios 2 (ethanol, LA) and 5 (methanol) had the highest profitability, while the former was the most robust scenario considering product price fluctuation, benefiting from multi-product revenues. LCA modelling has demonstrated that biomass cultivation played an important role in the environmental burden which demonstrated the importance of sustainable agricultural management on bio-based chemical production. Furfural production through a biphasic process contributed significantly to the environmental burdens (in all impact categories), which revealed the necessity of investigating other technologies to improve the yield of furfural, while avoiding solvent consumption. Gasification scenarios offered substantial environmental benefits over biochemical pathways due to lower levels of chemical consumption. Sustainable development of sugar industry could be moved forward by annexing 2G biorefinery into existing sugar mill to utilise lignocellulosic residues for production of some specific biofuels/biochemicals.

## Additional file

**Additional file 1: Table S1.** The main parameters implemented for the Aspen simulation of biorefinery scenarios. **Table S2.** Utilities condition of the developed biorefinery scenarios. **Table S3.** The specific energy consumption of the studied biorefinery scenarios (data of Fig. 4). **Table S4.** The detailed sales of the studied scenarios (data of Fig. 6). **Table S5.** Inventory for sugarcane cultivation. **Table S6.** Inventory for sugarcane processing in the sugar mill. **Table S7.** Inventory for the investigated biorefinery scenarios (per 65 ton/h biomass processed). **Table S8.** LCIA profiles of bioethanol production (unit: 1 ton bioethanol; method: CML-IA baseline V3.02). **Table S9.** LCIA profiles of bioethanol and lactic acid production (unit: 1 ton Lactic acid; method: CML-IA baseline V3.02). **Table S10.** LCIA profiles of bioethanol and furfural production (unit: 1 ton Furfural; method: CML-IA baseline V3.02). **Table S11.** LCIA profiles of butanol production (unit: 1 ton butanol; method: CML-IA baseline V3.02). **Table S12.** LCIA profiles of methanol production (unit: 1 ton methanol; method: CML-IA baseline V3.02). **Table S13.** LCIA profiles of FT syncrude production (unit: 1 ton FT syncrude; method: CML-IA baseline V3.02). **Table S14.** LCIA profiles for comparison of biorefinery scenarios (method: CML-IA baseline V3.02). **Table S15.** LCIA profiles for comparison of different scenarios for production of 1 tonne bioethanol (method: CML-IA baseline V3.02). **Table S16.** LCIA profiles for comparison of biorefinery scenarios (method: EI 99H). **Figure S1.** A schematic diagram of carbon flows through a biorefinery scenario (from sugarcane to final products.

## Abbreviations

ABE: acetone–butanol–ethanol
aLCA: attributional life cycle assessment
ALO-G: allothermal gasification
BRICS: Brazil, Russia, India and China
CHP: combined heat and power
CML: Centre of Environmental Science
DM: dry matter
EtOH: ethanol
EU: European Union
FT: Fischer–Tropsch
GHG: greenhouse gas
GWP_100_: global warming potential
IRR: internal rate of return
LA: lactic acid
LCA: life cycle assessment
LCIA: life cycle impact assessment
LCI: life cycle inventory
ODP: ozone depletion
OPEX: operating costs
POCP: photochemical oxidation
R_3_N: trimethylamine
SA: South Africa
SScF: simultaneous saccharification and co-fermentation
SSF: saccharification and fermentation
TCI: total capital investment
THF: tetrahydrofuran
USA: United States of America
WWT: waste water treatment
1G2G: integrated of first and second generation
2G: second generation
