# Supplementary material for: Multi-product biorefineries from lignocelluloses: a pathway to revitalisation of the sugar industry?
Source: Biotechnol Biofuels. 2017 Apr 11;10:87. doi: 10.1186/s13068-017-0761-9 (PMC5387292; doi:10.1186/s13068-017-0761-9)
Supplement: Supplementary file 1 — Additional file 1: Table S1. The main parameters implemented for the Aspen simulation of biorefinery scenarios. Table S2. Utilities condition of the developed biorefinery scenarios. Table S3. The specific energy consumption of the studied biorefinery scenarios (data of Fig. 4). Table S4. The detailed sales of the studied scenarios (data of Fig. 6). Table S5. Inventory for sugarcane cultivation. Table S6. Inventory for sugarcane processing in the sugar mill. Table S7. Inventory for the investigated biorefinery scenarios (per 65 ton/h biomass processed). Table S8. LCIA profiles of bioethanol production (unit: 1 ton bioethanol; method: CML-IA baseline V3.02). Table S9. LCIA profiles of bioethanol and lactic acid production (unit: 1 ton Lactic acid; method: CML-IA baseline V3.02). Table S10. LCIA profiles of bioethanol and furfural production (unit: 1 ton Furfural; method: CML-IA baseline V3.02). Table S11. LCIA profiles of butanol production (unit: 1 ton butanol; method: CML-IA baseline V3.02). Table S12. LCIA profiles of methanol production (unit: 1 ton methanol; method: CML-IA baseline V3.02). Table S13. LCIA profiles of FT syncrude production (unit: 1 ton FT syncrude; method: CML-IA baseline V3.02). Table S14. LCIA profiles for comparison of biorefinery scenarios (method: CML-IA baseline V3.02). Table S15. LCIA profiles for comparison of different scenarios for production of 1 tonne bioethanol (method: CML-IA baseline V3.02). Table S16. LCIA profiles for comparison of biorefinery scenarios (method: EI 99H). Figure S1. A schematic diagram of carbon flows through a biorefinery scenario (from sugarcane to final products. [file 13068_2017_761_MOESM1_ESM.pdf]

Multi-product biorefineries from lignocelluloses: A pathway to revitalisation of the Sugar Industry?

Somayeh Farzad,*[a] Mohsen Ali Mandegari,*[a] Miao Guo,[b] Kathleen F. Haigh,[a] Nilay Shah[b] and Johann F. Görgens✉[a]

* Equal Contribution

✉ Correspondence: E-mail: jgorgens@sun.ac.za; Department of Process Engineering, Stellenbosch University, Stellenbosch 7600, South Africa

Supplementary Information: 16 Tables and 1 Figure

Table S1 The main parameters implemented for the Aspen simulation of biorefinery scenarios

Parameter	*Value*	*Parameter*	*Value*
**Pretreatment of Scenarios 1, 2 and 3**		**Pretreatment of Scenario 4**	
Low pressure steam % wt of DM	10 %	Acid flow per biomass % wt	1.8 %
SO_2 % wt of DM	1.9 %	Cellulose→ Glucose	6.9 %
High pressure steam % wt of DM	50. %	Xylan→ Xylose	87.4 %
Cellulose→ Glucose	7.0 %	Xylan→ Furfural	5 %
Cellulose→ Acetic Acid	6.3 %	**SSF (Scenario 4)**	
Xylan→ Xylose	80.1 %	Cellulose→ Glucose	95.0 %
Xylan→ Furfural	3.1 %	Xylan→ Xylose	95.0 %
**SSCF (Scenario 1)**		Glucose → Butanol	55.7 %
Cellulose→ Glucose	74.5%	Glucose → Ethanol	6.6 %
Glucose → Ethanol	90.5 %	Glucose → Acetone	31.1 %
Xylose → Ethanol	76.6 %	Xylose → Butanol	20.1 %
Ethanol concentration % wt	5.5 %	Xylose → Ethanol	4.2 %
**Xylose fermentation Scenario 2**		Xylose → Acetone	18.5 %
Xylose → Lactic Acid	95.0 %	**Gasification (Scenarios 5 and 6)**	
**Xylose fermentation Scenario 3**		Feedstock split % wt of DM	42 %
Xylose → Furfural	83.0 %	Steam for gasification % wt of DM	100 %
**In common units**			
**Boiler unit**		**Steam and power unit**	
Deaerator pressure	3.3 atm	Number of extractions	3
Deaerator temperature	108 °C	Turbine isotropic efficiency	85 %
Boiler feed water pressure	62.2 atm	Mechanical efficiency	96 %
Boiler feed water temperature	176 °C	Sugar mill's steam extraction	27.6 atm
HHP steam pressure	62.2 atm	1st extraction pressure	13 atm
HHP steam temperature	452 °C	2nd extraction pressure	9.5 atm
Averaged burner temperature	870 °C	Condensate turbine pressure	0.1 atm
Combustion conversion	98 %	**Wastewater treatment unit**	
Inlet economizer temperature	278 °C	Wastewater temperature	35 °C
Air preheat temperature	185 °C	Chemical oxygen demand (COD)	16 g/L
Stack temperature	149 °C	Biological oxygen demand per COD	70 %
Boiler heat loss (total)	5%	Organic component → 3CH4 + CO_2	90 %
**Evaporation unit**		Methane to removed COD @ 25°C	0.23 kg/kg
First effect pressure	0.385 atm	Nutrient (per COD)	37 g/kg
LPS % of liquid inlet	5 %	COD of treated water	0.1 g/L
Second effect pressure	0.26 atm		
Third effect pressure	0.21 atm		
Forth effect pressure	0.16 atm		

Table S2 Utilities condition of the developed biorefinery scenarios

	Supplied		Return	
	Temperature (°C)	Pressure (atm)	Temperature (°C)	Pressure (atm)
Chilled water	4	2	15	1
Cooling water	28	2	37	1
Cooling air	30	1	35	1
High pressure steam (HPS)	266	13	192	12
Low pressure steam (LPS)	233	9.5	170	8
Sugar mill steam demand	338	27.6	122	2.2

Table S3 The specific energy consumption of the studied biorefinery scenarios (data of Figure 4)

	Cooling (kW/t*)	Heating (kW/t*)	Power (kW/t*)
Scenario 1: Ethanol	1198.8	1634.9	48.0
Scenario 2: Ethanol, LA	1166.5	1707.8	51.3
Scenario 3: Ethanol, Furfural	1599.9	2041.1	71.7
Scenario 4: Butanol	1873.7	822.3	580.6
Scenario 5: Methanol	620.6	61.8	335.4
Scenario 6: FTS	744.6	51.9	300.7

*: Specific energy consumption is defined as: kW of consumed energy per tonne feedstock to biorefinery (kw/t)

Table S4 The detailed sales of the studied scenarios (data of Figure 6)

	Sales (M $)											
	Total	Electricity	Ethanol	lactic acid	furfural	butanol	Formic acid	Acetic Acid	HMF	Acetone	methanol	FT syncrude
Scenario 1	58.58	3.9	54.7	0.0	0.0	-	-	-	-	-	-	-
Scenario 2	99.53	2.2	37.2	60.2	0.0	-	-	-	-	-	-	-
Scenario 3	54.03	4.1	28.5	0.0	16.4	-	0.8	3.5	0.8	-	-	-
Scenario 4	40.17	2.4	1.9	0.0	0.0	30.4	-	-	-	5.6	-	-
Scenario 5	56.5	0.3	0.0	0.0	0.0	-	-	-	-	-	56.2	-
Scenario 6	12.64	1.1	0.0	0.0	0.0	-	-	-	-	-	-	11.5

Table S5 Inventory for Sugarcane Cultivation*

Sugar cane agriculture	**Value**	**unit**
Cultivation area	400000	ha
Average cane harvest	60	t/ha
Irrigation water requirements	8000	m^3/ha
Electricity consumption for irrigation	108	kWh/ha
N_2O emissions from soil	1.5(1.25% of N input)	kg
NO_x emissions from soil	0.6(0.5% of N input)	kg
Fertilizer application rate/ha		
Urea, N	120	kg
Diammonium phosphate (DAP), P_2O_5	30	kg
Potassium oxide, KCl	125	kg
Herbicides use	1614(26.9g/ton sugar cane)	g
Herbicides loss in water bodies	3.228(0.2%)	g
Nitrogen loss in water bodies	161.4(10%)	g
Phosphorus loss in surface runoff/ha	1	kg
Pesticide use	132.6	g
Cane transportation		
Transportation, average distance, by:		
road(94% of cane)	25	km
rail(6% of cane)	50	km
Diesel consumption:		
Truck	1.08	MJ/tkm
rail	0.68	MJ/tkm
Fertilizers and herbicides transport distance	60	km

***Mashoko, L., Mbohwa, C., Thomas, V.M., 2010. LCA of the South African Sugar Industry. J. Environ. Plan. Manag. 53, 793–807**

Table S6 Inventory for Sugarcane processing in the sugar mill*

Sugar processing	
Sugarcane	6.0t
Bagasse produced	27.8% of cane
Molasses produced	4.1% of cane
Filter cake produced	6.8% of cane
Steam consumed/t of cane	520kg
Electricity consumption/t of cane	35kWh
Electricity generation/t of cane	150kWh
Coal consumption/t of cane	8.4kg
Water used for cane processing/t cane	0.6m3
Pollutant loadings of COD/t of cane	3320
Pollutant loadings of BOD5/t of cane	1590

*Mashoko, L., Mbohwa, C., Thomas, V.M., 2010. LCA of the South African Sugar Industry. J. Environ. Plan. Manag. 53, 793–807

Table S7 Inventory for the investigated biorefinery scenarios (per 65 ton/hour biomass processed)

Inputs (Feedstock and Chemicals)	EtOH	EtOH & Lactic acid	EtOH & Furfural	Butanol	Methanol	FT syncrude
**Bagasse and brown leaves (kg)**	6.50E+04	6.50E+04	6.50E+04	6.50E+04	6.50E+04	6.50E+04
**Sulphure**	2.77E+02	2.10E+02	2.43E+02	-	-	-
**Sulphuric acid (93%) (kg)**	4.00E-01	3.68E-01	3.07E-01	2.55E+02	-	-
**Ammonia (kg)**	3.00E+01	3.31E+01	1.37E+01	8.22E+02	-	-
**Diammonium phosphate (kg)**	4.40E+01	9.43E+01	3.75E+01	-	-	-
**Triethylamine (TEA) (kg)**	-	1.09E+02	-	-	-	-
**$MgSO_4$**	-	5.00E+01	-	-	-	-
**$Mg(OH)_2$**	-	5.50E+00	-	-	-	-
**Glucose (kg)**	7.54E+02	6.96E+02	6.38E+02	-	-	-
**Boiler & cooling tower Chemicals (kg)**	3.00E+02	3.00E+02	3.00E+02	3.00E+02	2.50E+02	2.50E+02
**NaoCl (kg)**	5.70E+00	5.06E+00	6.40E+00	3.60E+00	-	-
**Tetrahydrofuran (THF) (kg)**	-	-	6.43E+02	-	-	-
**NaCl (kg)**	-	-	2.00E+03	-	-	-
**HCl (kg)**	-	-	1.05E+03	-	-	-
**Outputs**						
**Ethanol production (kg)**	1.10E+04	6.73E+03	5.66E+03	3.53E+02	-	-
**Lactic acid production (kg)**	-	4.65E+03	-	-	-	-
**Furfural Production (kg)**	-	-	2.07E+03	-	-	-
**Acetic acid (kg)**	-	-	1.25E+03	-	-	-
**Formic acid (kg)**	-	-	2.28E+02	-	-	-
**Butanol production (kg)**	-	-	-	4.61E+03	-	-
**Aceton (kg)**	-	-	-	1.48E+03	-	-
**Methanol production (kg)**	-	-	-	-	1.28E+04	-
**FT syncrude production (kg)**	-	-	-	-	-	5.81E+03
**Exported electricity (MW)**	7.10E+00	5.60E+00	7.50E+00	4.30E+00	5.00E-01	1.80E+00
**Emissions and waste disposal**						
**EtOH (kg)**	9.82E-01	1.82E+01	4.24E+00	6.01E+00	-	-
**CO_2 (kg)**	8.99E+04	9.42E+04	8.89E+04	9.63E+04	5.87E+04	5.79E+04
**SO_2 (kg)**	1.63E+01	1.85E+01	7.60E+00	8.56E+00	-	-
**Acetic Acid (kg)**	6.43E+00	7.46E+00	1.69E+00	6.60E-01	-	-
**Furfural (kg)**	2.02E+00	1.96E+00	4.48E-01	2.77E-01	-	-
**CH_4 (kg)**	2.28E+00	2.28E+00	1.58E+00	-	-	6.42E+01
**NO_2 (kg)**	4.60E-02	4.60E-02	4.60E-02	4.60E-02	3.75E+00	3.08E+00
**CO**	3.00E-03	3.00E-03	3.00E-03	4.00E-03	7.76E+01	7.76E+01
**Acetone (kg)**	-	-	-	1.73E+02	-	-
**NO (kg)**	-	-	-	-	2.01E+02	1.66E+02
**C_4H_{10} (kg)**	-	-	-	-	-	5.47E+00
**Landfill disposal of ash (kg)**	3.25E+03	3.04E+03	3.10E+03	2.28E+03	7.88E+02	7.88E+02

Table S8 LCIA profiles of bioethanol production (unit: 1ton bioethanol; method: CML-IA baseline V3.02)

Impact category	Unit	Total	Pre-treatment	Enzyme production	SSCH	EtOH purification	Evaporation	Combustion	Waste water treatment	Biomass production	AD
**Abiotic depletion**	kg Sb eq	4.51E-04	1.90E-06	2.26E-05	6.10E-06	0.00E+00	0.00E+00	2.62E-05	2.20E-06	3.90E-04	2.14E-06
**Abiotic depletion(fossil fuel)**	MJ	1.93E+03	3.90E+02	9.87E+01	6.50E+01	0.00E+00	0.00E+00	3.76E+02	6.02E+00	9.95E+02	3.27E+00
**GWP_{100}**	kg CO_2 eq	4.57E+03	3.61E+00	9.10E+01	3.84E+00	5.21E+02	0.00E+00	3.87E+03	3.37E-01	8.24E+01	2.71E-01
**ODP**	kg CFC-11 eq	4.44E-05	4.11E-06	6.27E-06	7.79E-07	0.00E+00	0.00E+00	3.16E-06	6.87E-08	2.99E-05	1.27E-07
**Human toxicity**	kg 1,4-DB eq	1.19E+03	9.85E-01	2.06E+01	1.89E+00	1.84E-02	0.00E+00	1.03E+03	2.61E-01	1.39E+02	1.77E-01
**Fresh water eco-toxicity**	kg 1,4-DB eq	3.13E+02	4.26E-01	4.88E+00	3.51E-01	0.00E+00	0.00E+00	2.85E+02	7.27E-02	2.22E+01	1.26E-01
**Marine aquatic eco-toxicity**	kg 1,4-DB eq	3.86E+05	1.50E+03	4.52E+04	1.24E+03	0.00E+00	0.00E+00	2.59E+05	3.22E+02	7.80E+04	3.90E+02
**Terrestrial eco-toxicity**	kg 1,4-DB eq	9.75E-01	6.04E-03	1.29E-01	6.11E-03	0.00E+00	0.00E+00	6.66E-01	9.89E-04	1.66E-01	1.24E-03
**POCP**	kg C_2H_4 eq	1.57E-01	2.28E-03	2.09E-02	1.31E-03	1.64E-02	2.12E-02	2.34E-02	3.16E-02	3.95E-02	7.88E-05
**Acidification**	kg SO_2 eq	2.27E+00	3.86E-02	5.35E-01	2.77E-02	2.30E-01	0.00E+00	2.02E-01	6.38E-01	5.96E-01	1.88E-03
**Eutrophication**	kg PO_4^{3-} eq	2.26E+00	5.17E-03	4.59E-01	2.54E-03	1.36E-01	0.00E+00	1.37E+00	5.78E-04	2.77E-01	6.56E-04

Table S9 LCIA profiles of bioethanol and lactic acid production (unit: 1ton Lactic acid; method: CML-IA baseline V3.02)

Impact category	Unit	Total	Pre-treatment	Enzyme production	SSCH	EtOH purification	Evaporation	Combustion	Waste water treatment	Biomass production	AD	Lactic Acid production
**Abiotic depletion**	kg Sb eq	8.24E-04	2.32E-06	3.68E-05	8.91E-06	0.00E+00	0.00E+00	3.86E-05	3.47E-06	6.15E-04	3.00E-06	1.16E-04
**Abiotic depletion(fossil fuel)**	MJ	3.49E+03	4.66E+02	1.66E+02	9.49E+01	0.00E+00	0.00E+00	5.54E+02	9.48E+00	1.57E+03	4.57E+00	6.30E+02
**GWP_{100}**	kg CO_2 eq	7.41E+03	4.31E+00	1.34E+02	5.61E+00	5.67E+02	0.00E+00	6.54E+03	5.32E-01	1.30E+02	3.80E-01	2.39E+01
**ODP**	kg CFC-11 eq	6.99E-05	4.91E-06	9.38E-06	1.14E-06	0.00E+00	0.00E+00	4.65E-06	1.08E-07	4.71E-05	1.78E-07	2.40E-06
**Human toxicity**	kg 1,4-DB eq	1.79E+03	1.18E+00	3.07E+01	2.77E+00	1.09E-02	0.00E+00	1.52E+03	4.41E-01	2.19E+02	2.47E-01	1.83E+01
**Fresh water eco-toxicity**	kg 1,4-DB eq	4.70E+02	5.10E-01	7.26E+00	5.12E-01	0.00E+00	0.00E+00	4.20E+02	1.15E-01	3.51E+01	1.76E-01	5.75E+00
**Marine aquatic eco-toxicity**	kg 1,4-DB eq	5.97E+05	1.79E+03	6.63E+04	1.82E+03	0.00E+00	0.00E+00	3.82E+05	5.07E+02	1.23E+05	5.46E+02	2.11E+04
**Terrestrial eco-toxicity**	kg 1,4-DB eq	1.50E+00	7.22E-03	1.90E-01	8.92E-03	0.00E+00	0.00E+00	9.81E-01	1.56E-03	2.62E-01	1.73E-03	4.94E-02
**POCP**	kg C_2H_4 eq	7.86E-01	2.73E-03	3.08E-02	1.92E-03	1.15E-02	3.15E-01	5.30E-02	2.98E-01	6.23E-02	1.10E-04	1.11E-02
**Acidification**	kg SO_2 eq	3.87E+00	4.62E-02	7.87E-01	4.04E-02	1.37E-01	0.00E+00	3.78E-01	1.37E+00	9.40E-01	2.63E-03	1.64E-01
**Eutrophication**	kg PO_4^{3-} eq	3.29E+00	6.18E-03	6.68E-01	3.72E-03	1.08E-01	0.00E+00	2.03E+00	9.11E-04	4.37E-01	9.19E-04	4.10E-02

Table S10 LCIA profiles of bioethanol and furfural production (unit: 1ton Furfural; method: CML-IA baseline V3.02)

Impact category	Unit	Total	Pre-treatment	Enzyme production	SSCH	EtOH purification	Evaporation	Combustion	Waste water treatment	Biomass production	AD	Furfural production
**Abiotic depletion**	kg Sb eq	2.98E-03	2.74E-06	2.71E-05	7.54E-06	0.00E+00	0.00E+00	4.16E-05	3.66E-06	6.50E-04	4.01E-06	2.24E-03
**Abiotic depletion(fossil fuel)**	MJ	9.41E+03	5.70E+02	8.93E+01	8.03E+01	0.00E+00	0.00E+00	5.97E+02	1.00E+01	1.66E+03	6.12E+00	6.40E+03
**GWP_{100}**	kg CO_2 eq	7.91E+03	5.28E+00	1.25E+02	4.74E+00	4.72E+02	0.00E+00	6.76E+03	5.63E-01	1.37E+02	5.08E-01	4.00E+02
**ODP**	kg CFC-11 eq	1.40E-04	6.01E-06	8.24E-06	9.62E-07	0.00E+00	0.00E+00	5.02E-06	1.14E-07	4.98E-05	2.37E-07	6.99E-05
**Human toxicity**	kg 1,4-DB eq	2.11E+03	1.44E+00	2.76E+01	2.34E+00	8.47E-03	0.00E+00	1.64E+03	3.97E-01	2.31E+02	3.31E-01	2.11E+02
**Fresh water eco-toxicity**	kg 1,4-DB eq	6.48E+02	6.22E-01	6.60E+00	4.33E-01	0.00E+00	0.00E+00	4.53E+02	1.21E-01	3.71E+01	2.36E-01	1.50E+02
**Marine aquatic eco-toxicity**	kg 1,4-DB eq	1.07E+06	2.19E+03	6.27E+04	1.54E+03	0.00E+00	0.00E+00	4.12E+05	5.36E+02	1.30E+05	7.31E+02	4.58E+05
**Terrestrial eco-toxicity**	kg 1,4-DB eq	2.77E+00	8.83E-03	1.77E-01	7.54E-03	0.00E+00	0.00E+00	1.06E+00	1.65E-03	2.77E-01	2.32E-03	1.24E+00
**POCP**	kg C_2H_4 eq	4.35E-01	3.33E-03	2.84E-02	1.62E-03	7.11E-03	1.03E-01	1.94E-02	5.94E-02	6.59E-02	1.48E-04	1.46E-01
**Acidification**	kg SO_2 eq	5.49E+00	5.65E-02	7.32E-01	3.42E-02	1.06E-01	0.00E+00	3.12E-01	5.83E-01	9.94E-01	3.52E-03	2.67E+00
**Eutrophication**	kg PO_4^{3-} eq	4.26E+00	7.56E-03	6.44E-01	3.14E-03	5.73E-02	0.00E+00	2.18E+00	9.63E-04	4.62E-01	1.23E-03	8.99E-01

Table S11 LCIA profiles of butanol production (unit: 1ton butanol; method: CML-IA baseline V3.02)

Impact category	Unit	Total	Pre-treatment	SSF	Combustion	Seed generation	Biomass production
**Abiotic depletion**	kg Sb eq	1.16E-03	2.34E-04	2.66E-04	2.95E-05	9.07E-07	6.26E-04
**Abiotic depletion(fossil fuel)**	MJ	5.33E+03	4.68E+02	2.83E+03	4.23E+02	9.66E+00	1.60E+03
**GWP_{100}**	kg CO_2 eq	7.83E+03	2.28E+01	1.04E+03	6.63E+03	5.71E-01	1.32E+02
**ODP**	kg CFC-11 eq	9.09E-05	5.39E-06	3.39E-05	3.55E-06	1.16E-07	4.80E-05
**Human toxicity**	kg 1,4-DB eq	1.48E+03	1.60E+01	8.24E+01	1.16E+03	2.81E-01	2.23E+02
**Fresh water eco-toxicity**	kg 1,4-DB eq	3.78E+02	5.80E+00	1.53E+01	3.21E+02	5.21E-02	3.57E+01
**Marine aquatic eco-toxicity**	kg 1,4-DB eq	4.88E+05	1.66E+04	5.42E+04	2.92E+05	1.85E+02	1.25E+05
**Terrestrial eco-toxicity**	kg 1,4-DB eq	1.32E+00	4.13E-02	2.66E-01	7.49E-01	9.08E-04	2.66E-01
**POCP**	kg C_2H_4 eq	1.67E+00	1.32E-02	1.55E+00	4.01E-02	1.95E-04	6.35E-02
**Acidification**	kg SO_2 eq	3.44E+00	3.02E-01	1.20E+00	9.76E-01	4.11E-03	9.57E-01
**Eutrophication**	kg PO_4^{3-} eq	2.13E+00	2.93E-02	1.11E-01	1.55E+00	3.78E-04	4.45E-01

Table S12 LCIA profiles of methanol production (unit: 1ton Methanol; method: CML-IA baseline V3.02)

Impact category	Unit	Total	Gasification & Conditioning	Combustion	Biomass production
**Abiotic depletion**	kg Sb eq	3.89E-04	0.00E+00	7.81E-06	3.82E-04
**Abiotic depletion(fossil fuel)**	MJ	1.09E+03	0.00E+00	1.12E+02	9.76E+02
**GWP_{100}**	kg CO_2 eq	2.87E+03	5.33E+02	2.25E+03	8.07E+01
**ODP**	kg CFC-11 eq	3.02E-05	0.00E+00	9.41E-07	2.93E-05
**Human toxicity**	kg 1,4-DB eq	4.54E+02	0.00E+00	3.19E+02	1.36E+02
**Fresh water eco-toxicity**	kg 1,4-DB eq	1.07E+02	0.00E+00	8.50E+01	2.18E+01
**Marine aquatic eco-toxicity**	kg 1,4-DB eq	1.54E+05	0.00E+00	7.73E+04	7.64E+04
**Terrestrial eco-toxicity**	kg 1,4-DB eq	3.61E-01	0.00E+00	1.99E-01	1.63E-01
**POCP**	kg C_2H_4 eq	1.44E-01	9.84E-02	7.28E-03	3.87E-02
**Acidification**	kg SO_2 eq	5.43E+00	0.00E+00	4.85E+00	5.84E-01
**Eutrophication**	kg PO_4^{3-} eq	1.93E+00	0.00E+00	1.66E+00	2.71E-01

Table S13 LCIA profiles of FT syncrude production (unit: 1ton FT syncrude; method: CML-IA baseline V3.02)

Impact category	Unit	Total	Gasification & Conditioning	Combustion	FT synthesis	Biomass production
**Abiotic depletion**	kg Sb eq	4.49E-04	0.00E+00	9.00E-06	0.00E+00	4.40E-04
**Abiotic depletion(fossil fuel)**	MJ	1.25E+03	0.00E+00	1.29E+02	0.00E+00	1.12E+03
**GWP_{100}**	kg CO_2 eq	3.35E+03	6.15E+02	2.55E+03	9.56E+01	9.30E+01
**ODP**	kg CFC-11 eq	3.48E-05	0.00E+00	1.09E-06	0.00E+00	3.37E-05
**Human toxicity**	kg 1,4-DB eq	5.21E+02	0.00E+00	3.65E+02	0.00E+00	1.57E+02
**Fresh water eco-toxicity**	kg 1,4-DB eq	1.23E+02	0.00E+00	9.80E+01	0.00E+00	2.51E+01
**Marine aquatic eco-toxicity**	kg 1,4-DB eq	1.77E+05	0.00E+00	8.90E+04	0.00E+00	8.81E+04
**Terrestrial eco-toxicity**	kg 1,4-DB eq	4.16E-01	0.00E+00	2.29E-01	0.00E+00	1.87E-01
**POCP**	kg C_2H_4 eq	5.58E-01	1.13E-01	7.32E-03	3.93E-01	4.46E-02
**Acidification**	kg SO_2 eq	5.31E+00	0.00E+00	4.64E+00	0.00E+00	6.73E-01
**Eutrophication**	kg PO_4^{3-} eq	1.99E+00	0.00E+00	1.66E+00	1.77E-02	3.13E-01

Table S14 LCIA profiles for comparison of biorefinery scenarios (method: CML-IA baseline V3.02)

Impact category	Unit	EtOH	EtOH & Lactic acid	EtOH & Furfural	Butanol	Methanol	FT syntcrude
**Abiotic depletion**	kg Sb eq	8.82E-03	1.03E-02	3.65E-02	7.48E-03	7.86E-04	7.86E-04
**Abiotic depletion(fossil fuel)**	MJ	3.88E+04	4.47E+04	1.16E+05	5.19E+04	6.02E+03	6.02E+03
**GWP_{100}**	kg CO_2 eq	9.49E+04	9.76E+04	9.85E+04	9.99E+04	5.94E+04	6.03E+04
**ODP**	kg CFC-11 eq	8.81E-04	8.79E-04	1.71E-03	5.94E-04	5.76E-05	5.76E-05
**Human toxicity**	kg 1,4-DB eq	2.46E+04	2.34E+04	2.61E+04	1.64E+04	6.89E+03	6.85E+03
**Fresh water eco-toxicity**	kg 1,4-DB eq	6.48E+03	6.16E+03	8.05E+03	4.48E+03	1.86E+03	1.86E+03
**Marine aquatic eco-toxicity**	kg 1,4-DB eq	7.91E+06	7.76E+06	1.32E+07	4.86E+06	1.81E+06	1.81E+06
**Terrestrial eco-toxicity**	kg 1,4-DB eq	2.00E+01	1.96E+01	3.44E+01	1.42E+01	4.74E+00	4.74E+00
**POCP**	kg C_2H_4 eq	3.20E+00	1.03E+01	5.37E+00	2.08E+01	2.30E+00	9.53E+00
**Acidification**	kg SO_2 eq	4.63E+01	5.01E+01	6.76E+01	3.36E+01	1.04E+02	8.68E+01
**Eutrophication**	kg PO_4^{3-} eq	4.65E+01	4.30E+01	5.27E+01	2.23E+01	3.56E+01	3.14E+01

Table S15 LCIA profiles for comparison of different scenarios for production of 1 tonne bioethanol (method: CML-IA baseline V3.02)

Impact category	Unit	EtOH	EtOH & Lactic acid	EtOH & Furfural
**Abiotic depletion**	kg Sb eq	7.98E-04	6.76E-04	3.50E-03
**Abiotic depletion(fossil fuel)**	MJ	3.43E+03	2.87E+03	1.11E+04
**GWP_{100}**	kg CO_2 eq	8.09E+03	6.08E+03	9.29E+03
**ODP**	kg CFC-11 eq	7.86E-05	5.74E-05	1.65E-04
**Human toxicity**	kg 1,4-DB eq	2.11E+03	1.47E+03	2.48E+03
**Fresh water eco-toxicity**	kg 1,4-DB eq	5.55E+02	3.85E+02	7.62E+02
**Marine aquatic eco-toxicity**	kg 1,4-DB eq	6.83E+05	4.90E+05	1.25E+06
**Terrestrial eco-toxicity**	kg 1,4-DB eq	1.73E+00	1.23E+00	3.26E+00
**POCP**	kg C_2H_4 eq	2.77E-01	6.45E-01	5.11E-01
**Acidification**	kg SO_2 eq	4.02E+00	3.17E+00	6.45E+00
**Eutrophication**	kg PO_4^{3-} eq	3.99E+00	2.70E+00	5.00E+00

Table S16 LCIA profiles for comparison of biorefinery scenarios (method: EI 99H)

**Impact category**	**Unit**	**EtOH**	**EtOH & Lactic acid**	**EtOH & Furfural**	**Butanol**	**Methanol**	**FT syntcrude**
**Carcinogens**	DALY	7.08E-03	6.77E-03	8.60E-03	5.11E-03	2.04E-03	2.04E-03
**Resp. organics**	DALY	5.28E-06	2.01E-05	1.17E-05	4.40E-05	4.26E-07	5.39E-06
**Resp. inorganics**	DALY	4.21E-03	4.52E-03	7.55E-03	3.27E-03	1.86E-02	1.55E-02
**Climate change**	DALY	1.99E-02	2.05E-02	2.07E-02	2.10E-02	1.25E-02	1.27E-02
**Radiation**	DALY	8.04E-06	8.23E-06	2.48E-05	6.70E-06	9.49E-07	9.49E-07
**Ozone layer**	DALY	9.76E-07	9.75E-07	2.09E-06	6.21E-07	5.99E-08	5.99E-08
**Ecotoxicity**	PAF*m^2yr	5.56E+03	5.37E+03	7.50E+03	5.26E+03	1.58E+03	1.58E+03
**Acidification/Eutrophication**	PDF*m^2yr	1.28E+02	1.30E+02	2.00E+02	6.31E+01	1.18E+03	9.78E+02
**Land use**	PDF*m^2yr	8.08E+01	8.77E+01	1.26E+01	2.96E+01	1.19E+01	1.19E+01
**Minerals**	MJ surplus	8.67E+01	9.81E+01	3.81E+02	7.21E+01	1.20E+01	1.20E+01
**Fossil fuels**	MJ surplus	4.23E+03	4.93E+03	1.17E+04	6.58E+03	6.51E+02	6.51E+02

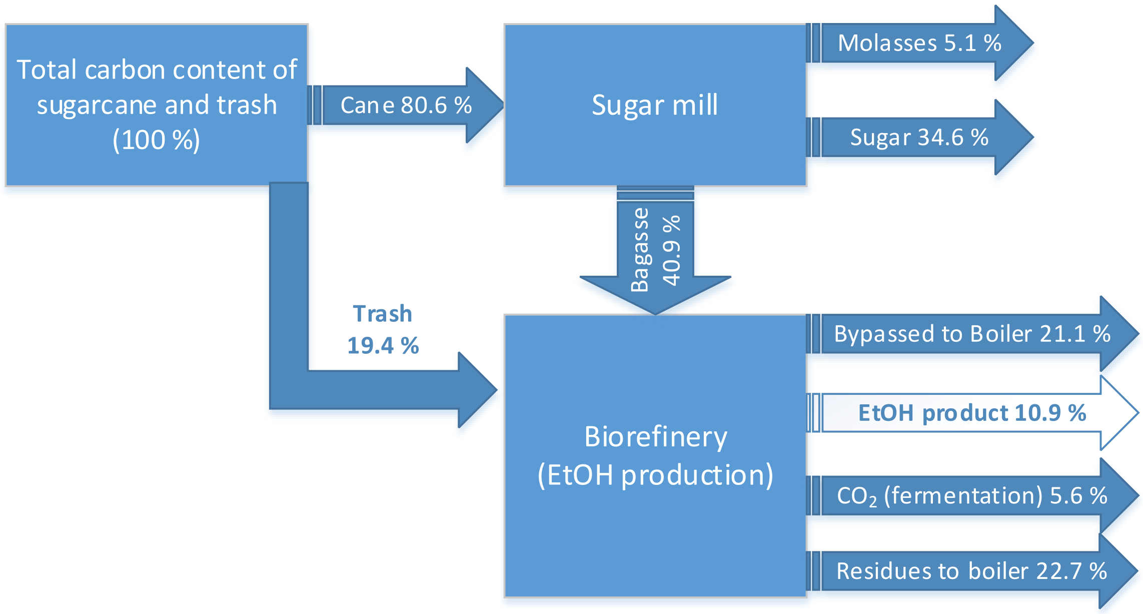

Figure S1 A schematic diagram of carbon flows through a biorefinery scenario (from sugarcane to final products).
